# Unbiased image segmentation assessment toolkit for quantitative differentiation of state-of-the-art algorithms and pipelines

**DOI:** 10.1186/s12859-023-05486-8

**Published:** 2023-10-12

**Authors:** Vishakha Goyal, Nick J. Schaub, Ty C. Voss, Nathan A. Hotaling

**Affiliations:** 1 Information Research Technology Branch (ITRB), National Center for Advancing Translational Science (NCATS), National Institutes of Health (NIH), 9800 Medical Center Dr, Rockville, MD 20850 USA; 2 Axle Research and Technologies, 6116 Executive Blvd #400, Rockville, MD 20852 USA

**Keywords:** Machine learning, Cell segmentation, Evaluation metrics

## Abstract

**Background:**

Image segmentation pipelines are commonly used in microscopy to identify cellular compartments like nucleus and cytoplasm, but there are few standards for comparing segmentation accuracy across pipelines. The process of selecting a segmentation assessment pipeline can seem daunting to researchers due to the number and variety of metrics available for evaluating segmentation quality.

**Results:**

Here we present automated pipelines to obtain a comprehensive set of 69 metrics to evaluate segmented data and propose a selection methodology for models based on quantitative analysis, dimension reduction or unsupervised classification techniques and informed selection criteria.

**Conclusion:**

We show that the metrics used here can often be reduced to a small number of metrics that give a more complete understanding of segmentation accuracy, with different groups of metrics providing sensitivity to different types of segmentation error. These tools are delivered as easy to use python libraries, command line tools, Common Workflow Language Tools, and as Web Image Processing Pipeline interactive plugins to ensure a wide range of users can access and use them. We also present how our evaluation methods can be used to observe the changes in segmentations across modern machine learning/deep learning workflows and use cases.

**Supplementary Information:**

The online version contains supplementary material available at 10.1186/s12859-023-05486-8.

## Introduction

As more cell segmentation methods are made available in literature [1–5], it is increasingly difficult to compare these methods on a given dataset using a comprehensive set of evaluation metrics. There are many evaluation metrics available [6–8], making it difficult to determine which metrics to use when evaluating a segmentation pipeline on a particular data set. Distinct types of evaluation metrics have their own limitations [9], making it essential to obtain results from multiple evaluation metrics to better understand cell segmentation quality. While the region of interest (ROI) level evaluation for segmentations can be used to calculate over and under segmentation [10, 11], the pixel level (PL) scores can be used to quantify the foreground and background detection [12] and fine grain differences between segmentations. Scores that evaluate feature histograms containing morphological, intensity, or textural features can be used to understand how meaningful the ROI and PL metrics are to measured values. To simplify the task of obtaining such metrics, there is a need to develop tools to assess each feature scalably so that images and segmentations of any size can be assessed. Additionally, multi-step analysis pipelines are needed to calculate them automatically and select the relevant features to compare different imaging algorithms.

Previous works have tackled the task of evaluating image segmentation methods using a variety of techniques. Some works have focused primarily on PL metrics [12], while others have focused on ROI metrics by implementing a cell-to-cell comparison approach [11]. Some researchers have also tried to combine intensity features and ROI level scores [13]**.** All these approaches of segmentation analysis are important; however, it can become challenging to implement an array of these metrics for a researcher trying to evaluate their model. It is common to evaluate models using one or two metrics at ROI or PL [14, 15], since it is difficult to implement a multitude of such metrics from scratch. The lack of an open-source pipeline to generate a variety of metrics was the impetus for this work. Here, three evaluation methods and several analysis pipelines are provided to the community which not only generate a comprehensive list of different metrics but also shortlist the relevant metrics using these tools, giving the researcher the means to select data driven metrics rather than choosing a metric commonly used in literature.

To evaluate segmentation metrics, a comprehensive comparison of image segmentation pipelines is performed in both 2D nucleus and cytoplasm images. Two structures were chosen to highlight the versatility of the proposed pipeline/tools and their applicability to be used on both simple (elliptical nuclei) and complex (cytoplasm) regions [16]. The complexity of cytoplasm morphology and texture highlights the need to quantify segmentations from multiple available assessment methods, as different metrics can show different facets of segmentation quality across the complex concave/convex regions. We also extract multiple features from the ground truth and predicted images and compare them using different distribution metrics. This comparison of features provides a better understanding of the segmentation quality by assessing biologically relevant parameters like mean intensity and solidity. The algorithms used for comparison in this paper are: UF-UNet [17], Mesmer [18]**,** SplineDist [19]**,** Allen Institute Cell Segmentation Tool (AICS) [20] and CellPose [21]. We also compare these methods against Columbus [22]**,** a commonly used proprietary commercial imaging tool. It is through comparing the above-mentioned state-of-the-art methods that we demonstrate the relevance and utility of creating pipelines to obtain comprehensive evaluation metrics to delineate and select optimal models based on identifying metrics that best separate them. The approach outlined in this manuscript is meant to provide an exhaustive, non-hypothesis driven assessment of model performance across three distinct concepts of model performance (pixel-wise, region-wise, and feature-wise). By assessing this comprehensive set of metrics (regardless of downstream application of the output of various sets of models) the Authors believe they remove bias from the assessment of model performance (not bias in the results of that assessment). For example, if a model has been pretrained on data from the same domain as the test set and is compared to another model that has not, it is unlikely that across all metrics outlined here the second model would perform “better” than the first. The proposed tools and pipeline are not meant to eliminate or estimate, in any way, the extent of bias in the above mentioned experimental set-up. Instead, they are meant to provide a comprehensive set of metrics that can assess differences between the results the two models generate and also identify the most significant contributors to the difference in performance between algorithms. What researchers do with this knowledge and the significance of these metrics to the underlying objective of the segmentation is still left to the expertise of those conducting the experiment.

## Methods

### Segmentation comparison pipeline

**Fig. 1:**
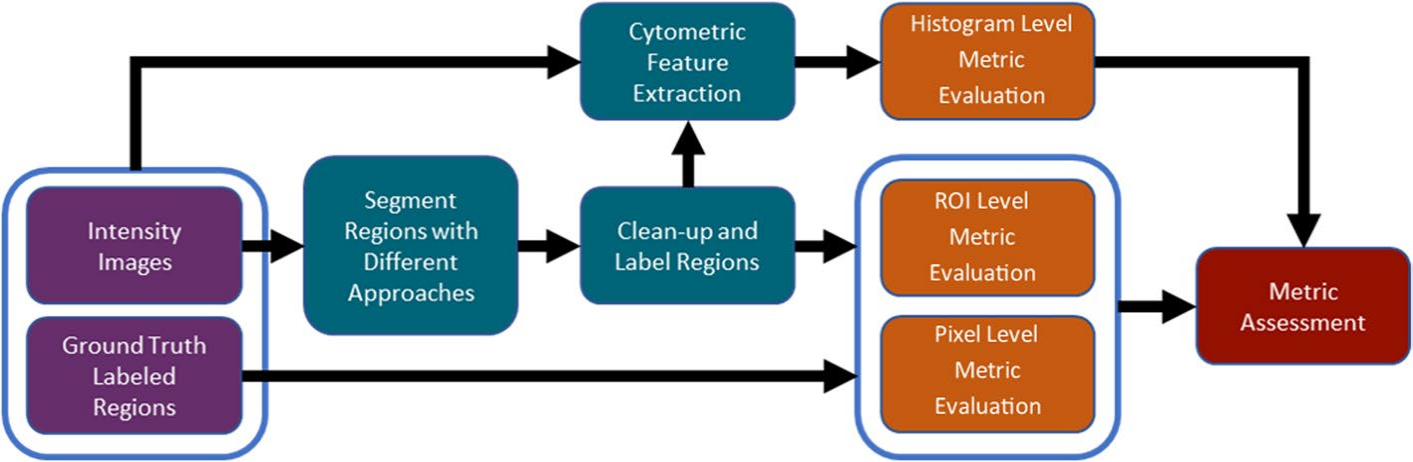
Overview of analysis pipeline and segmentation approaches. Intensity images from the fluorescent microscopy data set TissueNet, with ground truth labeled regions, were processed to evaluate Histogram, ROI, and Pixel level features. The first blue box shows that the intensity and ground truth images are inputs to the Cytometric Feature Extraction plugin and the second blue box shows that ROI and Pixel Level Evaluation Plugins have the same input of label and ground truth images. The input to Histogram (Feature) Evaluation plugin is the feature list from the cytometric feature extraction plugin

The pipeline that was used for evaluating segmented images using a multitude of evaluation metrics can be seen in Fig. 1. To promote reproducibility and numerical stability [23] all segmentation algorithms/pipelines in Table 1 were containerized and executed using Polus and the Web Image Processing Pipeline (WIPP) [24]. Polus-WIPP allowed for the creation of complex imaging pipelines with the help of interoperable analysis plugins, which include machine learning and deep learning models and other image processing algorithms. Containerization and execution in Polus-WIPP occurred for all approaches in Table 1 except Columbus X (v. 2.9.1) from Perkin Elmer. Columbus was chosen as an industry standard to compare against these pipelines. The Columbus workflow follows the same path from intensity images to segmentations, to segmentation clean-up as the AICS pipelines. Then, the segmentations are exported from Columbus as labeled bitmaps and the feature extraction and metric assessment steps are identical to those shown in Fig. 1. The test data was uploaded to Polus-WIPP, and all the segmentation and evaluation computations were run in the platform. The output from all the evaluation plugins was then visualized in Python using violin plots from the Matplotlib library 3.4.0 [25]. Polus-WIPP was used to execute all of the steps in the pipeline shown in Fig. 1, across all approaches listed in Table 1. The containerized plugins used for each step can be seen in Additional file 3: Table S1, along with links to their source code, docker container, and specific version used for this publication. Table 1 below compares the different segmentation plugins/pipelines, indicates if they were fine-tuned on TissueNet, and if the algorithms utilize a deep learning approach or not. Python v 3.7.3 was used for all libraries and packages in this manuscript unless otherwise indicated. While the clean-up step is not necessary, it is commonly used while segmenting images, especially when the methods being compared are not solely ML based, but also include commercial imaging tools, where the entire pipeline is created manually. A total of 69 metrics were generated from the plugins. These metrics were obtained by researching literature and finding commonly used metrics that can be used for comparing ground truth and segmented images.

**Table 1:** A table of all region segmentation approaches, whether they use deep learning or not, and whether models were fine-tuned on TissueNet or not that were assessed in this work

	Nuclear segmentation		Cytoplasm segmentation	
	Deep learning	Fine-tuned	Deep learning	Fine-tuned
Mesmer	Yes	Yes	Yes	Yes
AICS	No	No	No	No
SplineDist	Yes	Yes	N/A	N/A
UF-UNet	Yes	Yes	N/A	N/A
CellPose	Yes	No	Yes	No
Columbus	No	No	No	No

N/A means that the models were not used for cytoplasm segmentation

### Datasets assessed

The TissueNet v1.0 dataset (referred to as TissueNet in this manuscript) is composed of nuclear and cell (whole-cell and cell membrane) images provided by Greenwald, Miller et al. [18]. It consists of training images of 512 × 512 pixels, and validation and test images of 256 × 256 pixels. The dataset can be divided into 6 tissue types—Breast tissue, Gastrointestinal tissue, Immune cells, Lung, Pancreas and Skin Tissue. These images were captured using 6 different platforms—Codex, CyCif, IMC, Mibi, Mxif and Vectra. Since this dataset consists of a variety of tissue types and has publicly provided annotations, it was used for benchmarking the various segmentation methods.

### Segmentation algorithms

Segmentations of cell nuclei and cytoplasm were performed using the approaches indicated in Table 1 on the TissueNet dataset. After segmentation, evaluation metrics for each models’ predictions, independently on cytoplasm and nucleus, were assessed. Comparisons of models that were trained on more “general” cell nuclei and cytoplasm data to models that were further fine-tuned on TissueNet training data were also made (as shown in Table 1). The ImageJ UF-UNet plugin from University of Freiburg [17] was converted to python and the provided pre-trained weights, 2D Cell Net (v0), for the model were used for nuclear segmentation. The default model, initialized with 2D Cell Net (v0), was then fine-tuned on TissueNet nuclear images. Community provided pre-trained models were used for CellPose and SplineDist (versions 0.6.5 and 09/22/21, respectively). SplineDist was also fine-tuned on the TissueNet dataset for nuclear segmentation. Preliminary work to train and fine-tune SplineDist and UF-UNet on cytoplasm data was also performed but results were so poor that the training expense was determined to be too high to continue (data not shown). The AICS pipelines used were not originally created for nucleus or cytoplasm segmentation. The Nucleophosmin, Playground_npm1, pipeline was used for nucleus segmentation and the Sec61 beta and Lamin B1 pipeline, playground_curvi, was used for cytoplasm segmentation [26, 27]. Mesmer [18] from Deepcell library v. 0.10.0, was provided to the community pre-trained on TissueNet for Nuclear and Whole-cell images, and was containerized and used directly in this work. All plugin repositories, docker container locations, and CWL Tool (CLT) locations can be seen in Additional file 3: Table S1.

### Segmentation comparison methods

#### ROI level evaluation

ROI level evaluation is a comparison of each region of interest from a segmentation prediction to its associated region of interest in the ground truth. This technique can be used to observe over segmentation (e.g., multiple predicted regions for one ground truth region) and under segmentation (e.g., one predicted region for multiple ground truth regions). Distance between centroids of individual regions is used to match the ground truth with predicted regions [11]**.** Each ground truth ROI is matched with predicted ROIs. If the distance between a predicted ROI centroids is less than half the minor feret diameter of the ground truth ROI, it is counted as a match. A label (True Positive, False Positive, or False Negative) is assigned, and a pseudo confusion matrix is tabulated. No True Negative labels were assigned in this analysis since all background pixels are considered a single “negative object”. An ROI is labeled as a True Positive when exactly one predicted region matches the ground truth region. An ROI is labeled a False Positive when a predicted region matches with no ground truth region. An ROI is labeled a False Negative when a ground truth region matches with no predicted region. Once all ground truth regions have been compared with predicted regions, the matched predicted regions are deleted from the list of predicted ROIs and the remaining predicted ROIs are counted as false positives. Regions are only counted if their size is greater than two pixels. From the pseudo confusion matrix, a variety of metrics (not utilizing true negative) can be calculated. Additional file 2: File 1 provides formula for all ROI based metrics. In addition to these metrics, if a single ground truth region matches with multiple predicted regions, it is counted as an over-segmented region. Similarly, if multiple ground truth regions match with the same predicted region, they are counted as under-segmented.

#### Pixel level evaluation

This technique involves comparing each pixel from a predicted segmentation with each pixel from the corresponding ground truth segmentation. For these metrics, only a binary segmentation mask is needed in which regions of interest are assigned a value of 1, to represent foreground, and all other pixels are assigned a value of 0, to represent background. For each class of region (nucleus and cytoplasm) an independent comparison is made. Each pixel is assigned a label (True Positive, False Positive, True Negative, or False Negative), and a confusion matrix is tabulated. A pixel is labeled as a True Positive when both the predicted pixel and the ground truth pixel have a value of 1. A pixel is labeled as a False Positive when a predicted pixel has a value of 1 but the ground truth pixel has a value of 0. A pixel is labeled a True Negative when both predicted and ground truth pixels have a value of 0. A pixel is labeled a False Negative when the predicted region has a value of 0 and the ground truth pixel has a value of 1. Of note is that more pixel level metrics are assessed than region level. This is due to pixel level metrics having a full confusion matrix, with true negatives, to be able to calculate metrics from. Additional file 2: File 1 provides formula for all pixel level metrics.

#### Feature level evaluation

After segmentation, 367 cytometric features were extracted from regions using the Nyxus library [28] v. 0.2.4, for both nuclei and whole cell regions. After extraction, feature values were binned into histograms using Freedman-Diaconis rule and 38 distribution comparison metrics were used to evaluate the changes in the distributions of the extracted features between ground truth and predicted images. A subset of the total features (area, perimeter, mean intensity, and solidity) is presented in the results section to compare the segmentations with respect to cell morphology and intensity. 9 features and 3 distribution metrics per feature can also be found in Additional file 1: Figs. S65–S118. Out of the 38 feature level metrics assessed; histogram intersection was selected to compare the extracted features. It was found, for this dataset, nearly all feature level metrics were highly correlated for a given feature and thus the results shown for histogram intersection were reproducible across all feature level metrics extracted. For the scope of this paper, 16 distance metrics out of the 38 feature metrics were used for PCA analysis for feature selection. The feature extraction plugin generates 367 features per image and with 38 metrics per feature, it generates 13,946 metrics per image pair. While certainly obtainable for this study, since the point of this paper was not to perform an exhaustive analysis but instead to show a method and tools that enables that exhaustive method, the Authors chose to limit their feature space. The distance metrics are as follows—L1 distance, L2 distance, L infinity, Kolmogorov–Smirnov Divergence, Match Distance, Cramer-von Mises Distance, PSI value, Kullback–Leibler Divergence, Jensen Shannon Distance, Histogram Intersection, Correlation, Chi Square, Bhattacharya Distance, Cosine Distance, Canberra Distance and Wasserstein Distance. Additional file 2: File 1 provides formula for all feature level metrics. 21 out of the 38 feature metrics were implemented using the forecasting metrics GitHub repository from Boris Shishov [29]. The code was first accessed in November 2021.

### Dimension reduction and feature importance

The principal component analysis (PCA) function from SciKit Leearn v 1.0.1 was used to generate Eigen vectors and values, as well as assessing data variance. The CatBoost library was implemented from the library version 1.0.2, https://github.com/catboost/catboost. The CatBoost model was trained using 500 iterations and a learning rate of 0.01. For both PCA and Catboost analysis, only the metrics with the highest importance/weight were discussed in this paper.

### Statistics

Normality of metrics was assessed using the Jarque Bera test and the tseries package v 0.10-49. All metrics were found to be significantly non-normal, so the Freidman test using stats package v 4.1.2, followed by Tukey's Post Hoc test was used to generate pairwise comparisons of the metrics from different models for all plots. Population marginal means, as opposed to least square means, were used for the linear model, and the emmeans package, v 1.7.2, in R was used with Tukey adjustment to generate the pairwise p-values between different models. The effective significance level calculated with Tukey adjustment was *p* < 0.0001, meaning results were determined to be statistically significant when *p* < 0.0001. The histogram intersection of area was Null for a small set of images where no regions were found, and thus no distributions were calculated (< 10% of total), therefore, for these metrics only common images across all segmentation methods without null values were used for the statistical analysis. This analysis was used in conjunction with the mean values of metrics to understand the model performance individually and with respect to each other.

## Results

### Segmentation comparison

**Fig. 2:**
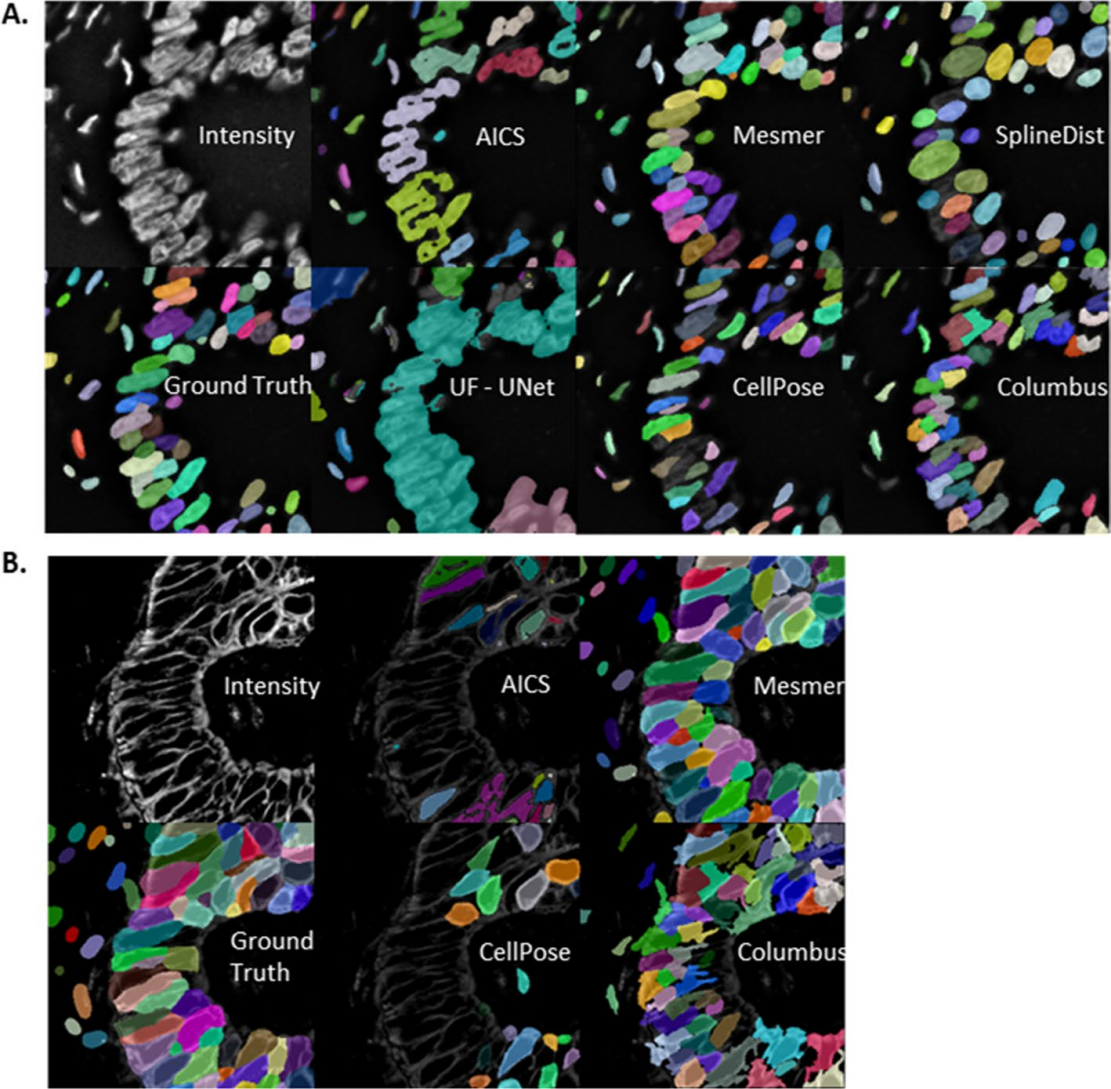
Segmented Labels from pre-trained and commercial imaging algorithms. **A** Nuclear Segmentation Labels. **B** Cytoplasm Segmentation Labels

The goal of this work was to compare segmentation metrics and find a set of metrics that characterizes various aspects of segmentation quality. To that end, multiple segmentation pipelines were run on TissueNet. The only model tuned to TissueNet was Mesmer, so the expectation would be that Mesmer outperforms all other models on TissueNet segmentation. The segmentation masks from different imaging algorithms can be seen in Fig. 2A for nuclear images and Fig. 2B for cytoplasmic images across all segmentation methods. Quantitative assessment of each of these segmentation approaches was performed and described below.

#### ROI level metrics

**Fig. 3:**
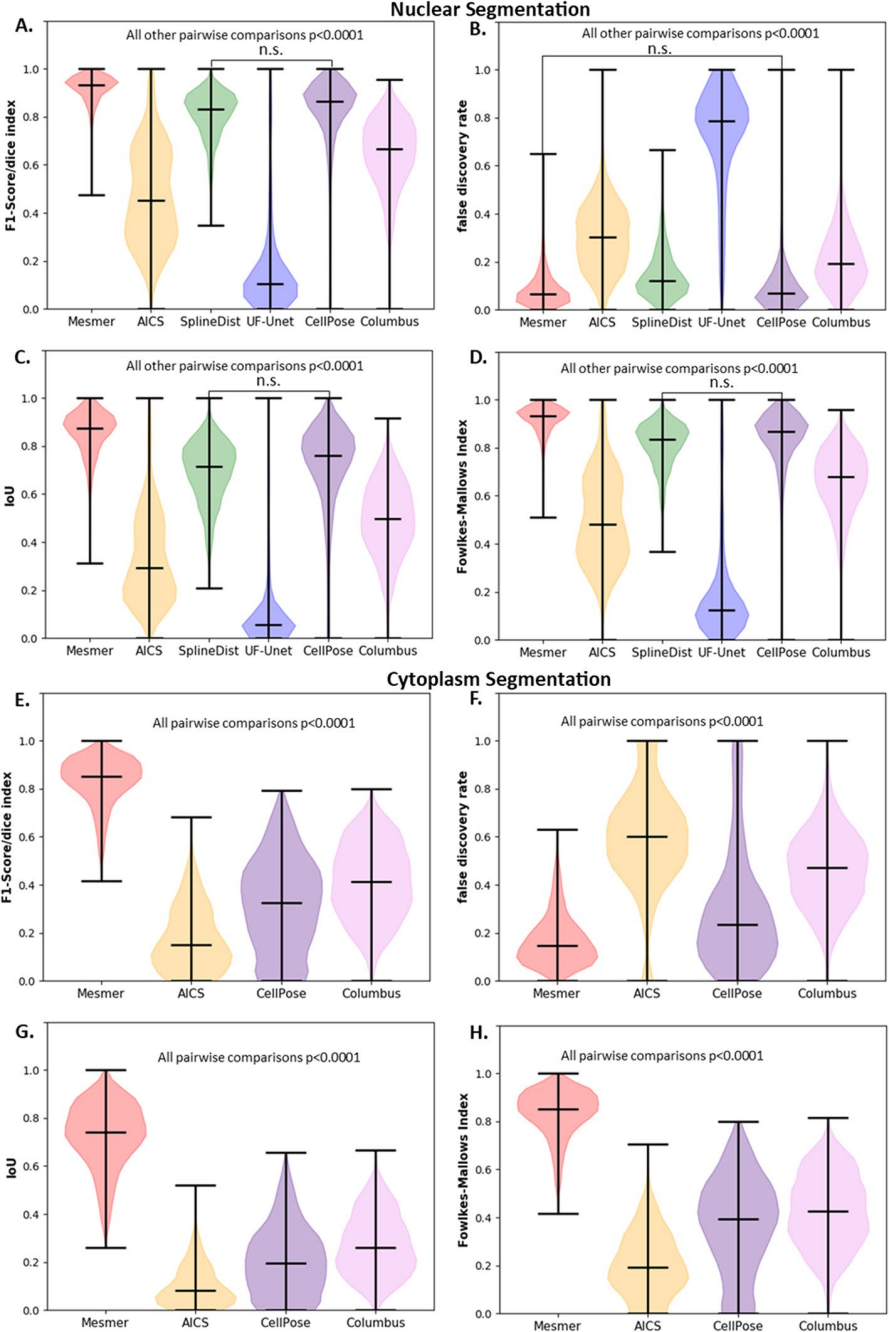
ROI level metrics for nucleus and cytoplasm regions. Nuclear Segmentation (**A**). F1-Score (**B**). False Discovery Rate (**C**). Intersection over Union (IoU) (**D**). Fowlkes-Mallows Index. Cytoplasm Segmentation **E**. F1-Score **F**. False Discovery Rate **G**. IoU **H**. Fowlkes-Mallows Index. n.s.—not significant

ROI level metrics measure the accuracy of individual ROIs, and ROI metrics from Mesmer segmentations were expected to outperform other segmentation pipelines since Mesmer was trained on the reference data set (TissueNet). Table 2 shows the mean ± std for performance metrics for nuclear and cytoplasm segmentation at the region level and Fig. 3 shows the violin plots for these metrics. While Mesmer was significantly better with nuclear segmentation than other models for F1 score (Fig. 3A), it did not perform the best for false discovery rate (FDR, Fig. 3B). Mesmer was also found to perform other models for Intersection over Union (IoU) (Fig. 3C), and Fowlkes–Mallows index (FMI) (Fig. 3D). Unexpectedly, the FDR for Mesmer was not found to be significantly different than that of CellPose, despite significantly outperforming them for other metrics. Thus, all metrics showed Mesmer outperformed all other models except for the FDR.

**Table 2:** Mean ± std for ROI based metrics using default models for nuclear and cytoplasm segmentations

Method	Region	F1 Score	IoU score	False discovery rate	Fowlkes–Mallows index
Mesmer	Nucleus	**0.91 ± 0.06**	**0.85 ± 0.09**	**0.08 ± 0.07**	**0.91 ± 0.06**
CellPose	Nucleus	0.81 ± 0.16	0.71 ± 0.18	0.09 ± 0.11	0.82 ± 0.15
SplineDist	Nucleus	0.81 ± 0.09	0.69 ± 0.12	0.14 ± 0.09	0.81 ± 0.09
Columbus	Nucleus	0.64 ± 0.14	0.49 ± 0.14	0.21 ± 0.12	0.66 ± 0.13
AICS	Nucleus	0.47 ± 0.20	0.33 ± 0.19	0.30 ± 0.13	0.50 ± 0.18
UF-UNet	Nucleus	0.16 ± 0.20	0.11 ± 0.17	0.72 ± 0.21	0.18 ± 0.19
Mesmer	Cytoplasm	**0.83 ± 0.09**	**0.72 ± 0.13**	**0.16 ± 0.10**	**0.83 ± 0.09**
CellPose	Cytoplasm	0.32 ± 0.19	0.20 ± 0.14	0.29 ± 0.24	0.37 ± 0.19
SplineDist	Cytoplasm	N/A	N/A	N/A	N/A
Columbus	Cytoplasm	0.41 ± 0.15	0.27 ± 0.12	0.46 ± 0.15	0.42 ± 0.15
AICS	Cytoplasm	0.17 ± 0.13	0.10 ± 0.08	0.59 ± 0.21	0.20 ± 0.12
UF-UNet	Cytoplasm	N/A	N/A	N/A	N/A

Bold indicates best performer for a given metric

Comparison of the remaining segmentation pipelines revealed stark differences in performance between cytoplasm and nuclear segmentation. CellPose and SplineDist, had comparable performance for all metrics (for cell nuclei) and were not statistically different for F1 score, IoU and FMI. For cell nuclei Columbus performed worse across all metrics than Mesmer, CellPose, or SplineDist but outperformed AICS and UF-UNet. While for cell cytoplasm segmentation (Fig. 3E–H) Columbus generally outperformed CellPose across all metrics, with the exception of FDR (Fig. 3F) where Columbus had a statistically higher prevalence of identifying false positive regions erroneously. Across all metrics the AICS pipeline performed better than UF-UNet but worse than all other approaches for both nuclei and cytoplasm. The UF-UNet model performed the worst across all metrics shown in Fig. 3A–D. Across all segmentation pipelines except Mesmer, the metrics present a comprehensive assessment that none of the models perform well in cell cytoplasm segmentation, and while nuclear segmentation was acceptable for several algorithms/pipelines without fine-tuning, Mesmer performed statistically better.

#### PL metrics

**Fig. 4:**
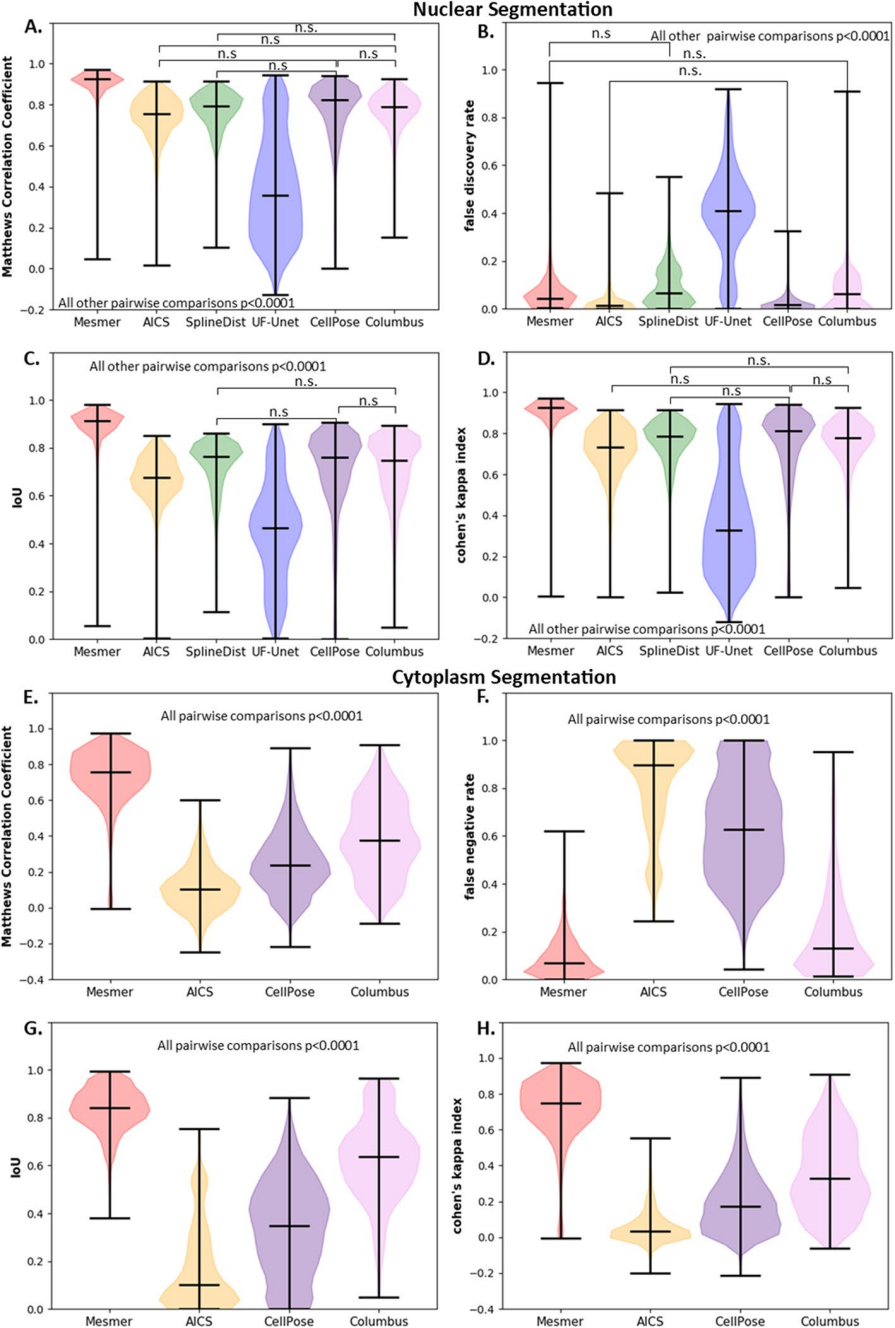
Pixel level metrics for nucleus and cytoplasm regions. Nuclear Segmentation **A** Matthews Correlation Coefficient. **B** False Discovery Rate. **C** IoU. **D** Cohen’s Kappa Index. Cytoplasm Segmentation. **E** Matthews Correlation Coefficient. **F** False Negative Rate. **G** IoU. **H** Cohen’s Kappa Index. n.s.—not significant

PL metrics give a better indication of total foreground pixels accurately segmented, and as with the ROI metrics, it was expected that Mesmer would outperform all other models. Table 3 shows the mean ± std for performance metrics for nuclear and cytoplasm segmentation at the pixel level and Fig. 4 shows the violin plots for these metrics. As expected, Mesmer performed significantly better on nuclear segmentation than other models for traditional metrics such as Matthew’s Correlation Coefficient (MCC) (Fig. 4A) and IoU (Fig. 4B). Unexpectedly, both CellPose and AICS had a significantly lower FDR (*p* < 0.0001, Fig. 4C) for nuclear segmentation than did Mesmer, meaning a lower prevalence of false positives. The FDR for Mesmer was not found to be significantly different from that of Columbus and SplineDist (*p* = 0.4852 and 0.01 respectively). AICS was found to have a lower FDR than Mesmer, as seen in Fig. 4C, despite having poorer segmentation. This can be attributed to AICS having the least number of pixel level false positives, as seen in Additional file 1: Fig. S21. Since Mesmer is trained on TissueNet, it was unexpected that it would have a higher false discovery rate than other segmentation algorithms. In agreement with ROI metric results, UF-UNet performed significantly worse than all other approaches. Interestingly, while Columbus generally performed worse than Mesmer, CellPose, and SplineDist for region level metrics (Fig. 3), it was not statistically different than CellPose or SplineDist when assessing most pixel level metrics.

**Table 3:** Mean ± std for pixel-based metrics using default models for nuclear and cytoplasm segmentations

Method	Region	IoU score	False discovery rate	Cohen’s kappa index	Matthews corr. coefficient
Mesmer	Nucleus	**0.88 ± 0.10**	0.08 ± 0.11	**0.89 ± 0.10**	**0.90 ± 0.09**
CellPose	Nucleus	0.69 ± 0.18	**0.02 ± 0.02**	0.73 ± 0.19	0.75 ± 0.17
SplineDist	Nucleus	0.72 ± 0.11	0.09 ± 0.08	0.75 ± 0.12	0.76 ± 0.10
Columbus	Nucleus	0.70 ± 0.14	0.07 ± 0.07	0.74 ± 0.13	0.76 ± 0.11
AICS	Nucleus	0.67 ± 0.09	0.02 ± 0.04	0.71 ± 0.13	0.74 ± 0.10
UF-UNet	Nucleus	0.45 ± 0.20	0.39 ± 0.18	0.37 ± 0.25	0.39 ± 0.25
Mesmer	Cytoplasm	**0.83 ± 0.09**	**0.08 ± 0.07**	**0.71 ± 0.17**	**0.73 ± 0.16**
CellPose	Cytoplasm	0.33 ± 0.19	0.63 ± 0.21	0.20 ± 0.17	0.25 ± 0.17
SplineDist	Cytoplasm	N/A	N/A	N/A	N/A
Columbus	Cytoplasm	0.62 ± 0.17	0.19 ± 18	0.35 ± 0.21	0.38 ± 0.21
AICS	Cytoplasm	0.17 ± 0.17	0.81 ± 0.18	0.06 ± 0.09	0.11 ± 0.13
UF-UNet	Cytoplasm	N/A	N/A	N/A	N/A

Bold indicates the top performer for a given region type for each metric shown

For cell cytoplasm segmentation (Fig. 4E–H) Mesmer performed better than all other algorithms and Columbus generally outperformed CellPose. The AICS pipeline performed the worst across all metrics shown in Fig. 4E–H. Figure 4 highlights that performance on Region Level features (Fig. 3) is not always predictive of performance at the Pixel Level and vise-a-versa. Thus, to pick optimal assessment metric(s) a nuanced understanding of the application is needed and cannot be directly inferred from performance of an algorithm at a given “level”.

#### Feature based metrics

**Fig. 5:**
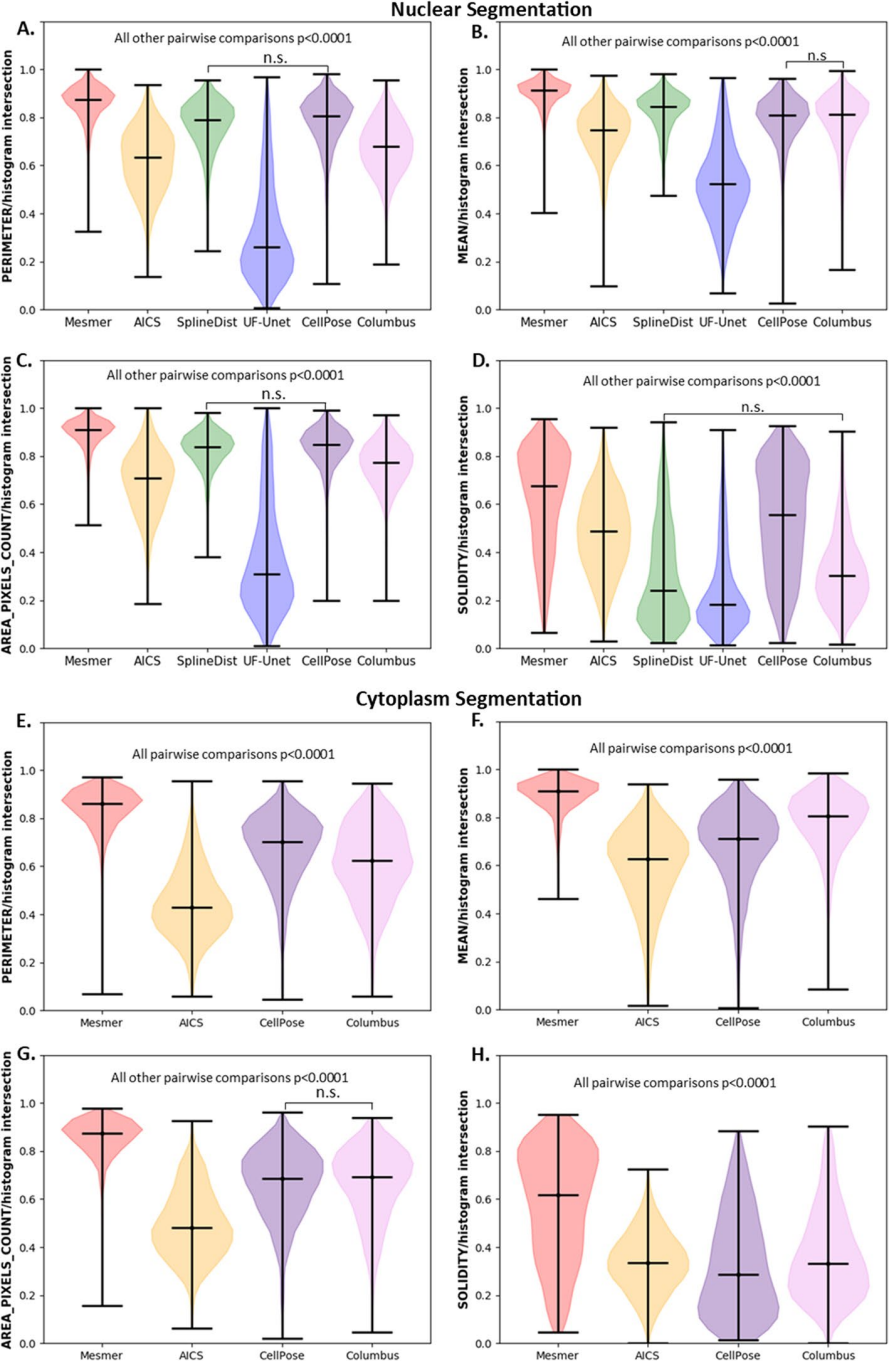
Histogram Intersection metrics on nucleus and cytoplasm regions. Nuclear Segmentation. **A** Perimeter. **B** Mean Intensity. **C** Area. **D** Solidity. Cytoplasm Segmentation. **E** Perimeter. **F** Mean Intensity. **G** Area. **H** Solidity. n.s.—not significant

Feature based metrics assess features extracted from ROIs and is an important set of metrics because differences in segmentation may not produce meaningful differences in features. Table 4 shows the mean ± std for performance metrics for nuclear and cytoplasm segmentation at the pixel level and Fig. 5 shows the violin plots for these metrics. As with previous sections, Mesmer was expected to have the best performance. As expected, Mesmer had a better histogram intersection metric for various features than other models for nuclear segmentation (Fig. 5A–D). Both CellPose and SplineDist performed similarly across most nuclear features, but SplineDist was the worst performer for some metrics (Fig. 5D). Generally, Columbus either performed equivalently (Fig. 5B) to CellPose or worse (Fig. 5A, C, D) depending on the nuclear metric assessed. AICS pipelines performed between UF-UNET (worst) and Columbus. Taken together Mesmer outperforms other models in nuclear feature level metrics with CellPose and SplineDist following behind. Also, in agreement with ROI and PL results, UF-UNet performed significantly worse than all other approaches.

**Table 4:** Mean ± std for feature-based metrics using default models for nuclear and cytoplasm segmentations

Method	Region	Intersection area	Intersection perimeter	Intersection mean intensity	Intersection solidity
Mesmer	Nucleus	**0.89 ± 0.06**	**0.85 ± 0.08**	**0.89 ± 0.05**	**0.62 ± 0.20**
CellPose	Nucleus	0.83 ± 0.09	0.77 ± 0.11	0.78 ± 0.12	0.53 ± 0.22
SplineDist	Nucleus	0.82 ± 0.07	0.77 ± 0.10	0.82 ± 0.08	0.30 ± 0.20
Columbus	Nucleus	0.76 ± 0.09	0.67 ± 0.11	0.78 ± 0.12	0.32 ± 0.15
AICS	Nucleus	0.69 ± 0.12	0.62 ± 0.13	0.73 ± 0.11	0.48 ± 0.16
UF-UNet	Nucleus	0.37 ± 0.22	0.32 ± 0.20	0.52 ± 0.16	0.24 ± 0.17
Mesmer	Cytoplasm	**0.85 ± 0.78**	**0.84 ± 0.08**	**0.89 ± 0.07**	**0.58 ± 0.22**
CellPose	Cytoplasm	0.66 ± 0.14	0.67 ± 0.14	0.67 ± 0.16	0.32 ± 0.20
SplineDist	Cytoplasm	N/A	N/A	N/A	N/A
Columbus	Cytoplasm	0.66 ± 0.15	0.61 ± 0.15	0.78 ± 0.12	0.36 ± 0.18
AICS	Cytoplasm	0.49 ± 0.14	0.44 ± 0.52	0.59 ± 0.16	0.33 ± 0.12
UF-UNet	Cytoplasm	N/A	N/A	N/A	N/A

Bold indicates the top performer for a given region type for each metric shown

For cytoplasm features (Fig. 5E–H), Mesmer performed better than all other segmentation pipelines and Columbus generally outperformed CellPose or was not statistically different. The AICS pipeline performed the worst for cytoplasm features across all metrics shown in Fig. 5E–H. For more complex morphological structures like cell cytoplasm, it is notable that different trends between model performance were seen than for simple structures such as the nuclei.

As mentioned at the beginning of this section, differences in segmentation may not lead to meaningful differences in extracted features and this is highlighted when comparing feature level metrics to ROI and PL metrics. Comparing ROI and PL metrics (Figs. 3 and 4 respectively) to feature level metrics (Fig. 5) shows that ROI and PL metrics are not necessarily predictive of feature level performance and vise-a-versa. A good example of this can be seen in Fig. 5B and Table 4, which shows that the mean intensity feature for Columbus and CellPose was not significantly different. However, CellPose showed significantly better performance than Columbus with respect to ROI metrics in “ROI level metrics” section, highlighting how Columbus had worse segmentation accuracy but similar feature accuracy to CellPose. Despite spot differences, looking across all metrics assessed, the feature level metrics were in line with the ROI and PL metrics, with Mesmer outperforming other methods and AICS performing the worst for whole-cell segmentation.

### Metric assessment as a function of network training for nuclear segmentation

**Fig. 6:**
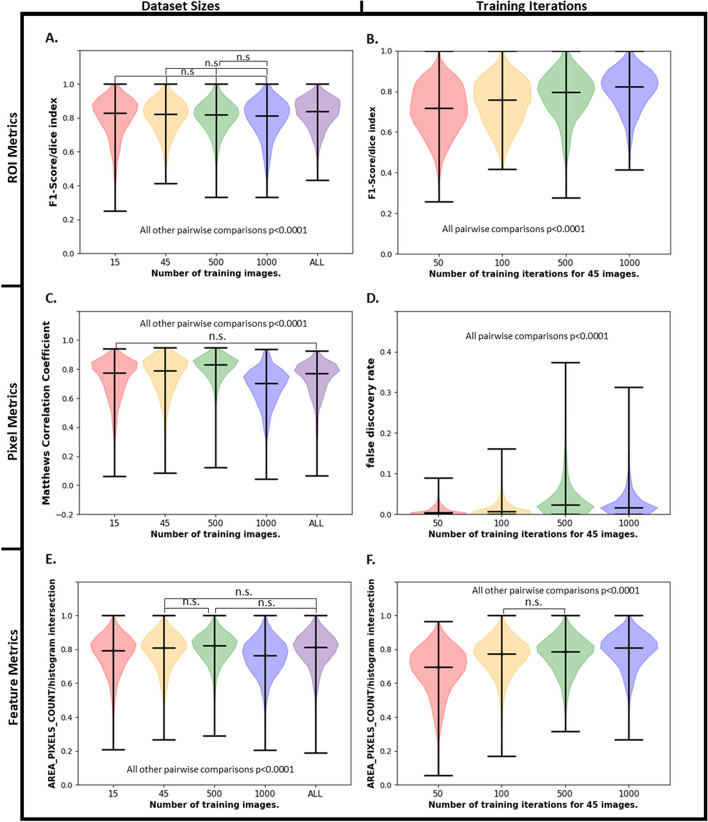
Assessment of relevant metrics as a function of training the UF-UNet model on TissueNet dataset. ROI Level F1 Score for different **A** Training Sizes and **B** Training iterations. Pixel level **C** MCC Score for different Training sizes and **D** FDR for different Training iterations. Feature level Histogram Intersection of Area for different **E** Training Sizes and **F** Training iterations. n.s.—not significant

Once the performance metrics were calculated on pretrained models, we then assessed the metrics after fine tuning image processing pipelines on TissueNet. We calculated metrics as a function of the number of images the model was fine-tuned on and number of epochs. UF-Unet was selected for testing since it performed worst on all metrics in the previous section, so it was expected that the largest changes would be observed when fine-tuning this model. Figure 6 shows ROI (Fig. 6A, B), PL (Fig. 6C, D), and feature (Fig. 6E, F) level metrics after training UF-UNet on different numbers of TissueNet nuclear images and different numbers of epochs. Figure 6A, C, E show the evolution of various evaluation metrics as a function of training dataset size; 15–2601 (all TissueNet training) images for a constant number of epochs. Figure 6B, D, F show how the model evaluation metrics evolved as a function of epoch for 50–1000 epochs. These comparisons were included to show the variation in segmentations with training parameters and highlight the importance of capturing a variety of evaluation metrics when comparing different versions of a model as well as how different metrics evolve as a function of training data and epoch. An additional 25 plots of metric values can be seen in Additional file 1: Figs. S119–S143. The mean ± std values for relevant metrics are included in Additional file 3: Tables S2–S7.

As expected, in Fig. 6A, ROI metrics significantly improved with using all available training data. The improvement in ROI metric performance was also visible in Fig. 6B when increasing the amount of training epochs. The F1 score was not found to be significantly different between n = 15, n = 45, n = 500 and n = 1000 images, but the F1 score was significantly different when all training images (n = 2601) were used (*p* < 0.0001). Figure 6A, B highlights F1 score, however, all other metric plots for ROI can be seen in Additional file 1: Figs. S119–S127. Although fine-tuning UF-Net improved the resulting segmentation metrics, which were significantly worse than Mesmer, fine-tuning UF-Net did not lead to significantly better performance than Mesmer.

The PL metrics also improved with respect to number of training iterations (Fig. 6C, D). While the ROI level scores in 6A and Additional file 1: Figs. S119–S124 improved with all training data, Fig. 6C and Additional file 1: Figs. S133–S134 show that PL metric scores plateaued at 500 images. Interestingly, the FDR metric for pixel level data did not improve as a function of number of training epochs (Fig. 6D), and unexpectedly smallest for 50 epochs. The discrepancies between ROI and PL metrics for training with different number of training images highlight the importance of calculating these metrics.

Figure 6E, F showed similar trends in feature metrics to that of PL metrics. The UF-Net area metric plateaued when fine-tuned with 500 images − 0.80 ± 0.09 (compared to the entire dataset score of 0.79 ± 0.10 which was not statistically different). There was no significant difference between the histogram intersection of area for n = 45, n = 500 and n = 2601 (all) images. The average histogram intersection of area metric improved for UF-UNet with more training, increasing from 0.67 ± 0.14 over 50 epochs to 0.79 ± 0.10 for 1000 epochs. While the ROI based metrics showed segmentations to significantly improved when fine-tuned on all training images, the PL and feature metric improvements plateaued at n = 500 images. These findings are seen across most metrics measured and can be seen in Additional file 1: Figs. S138–S140.

### Segmentation comparison with trained machine learning models

**Fig. 7:**
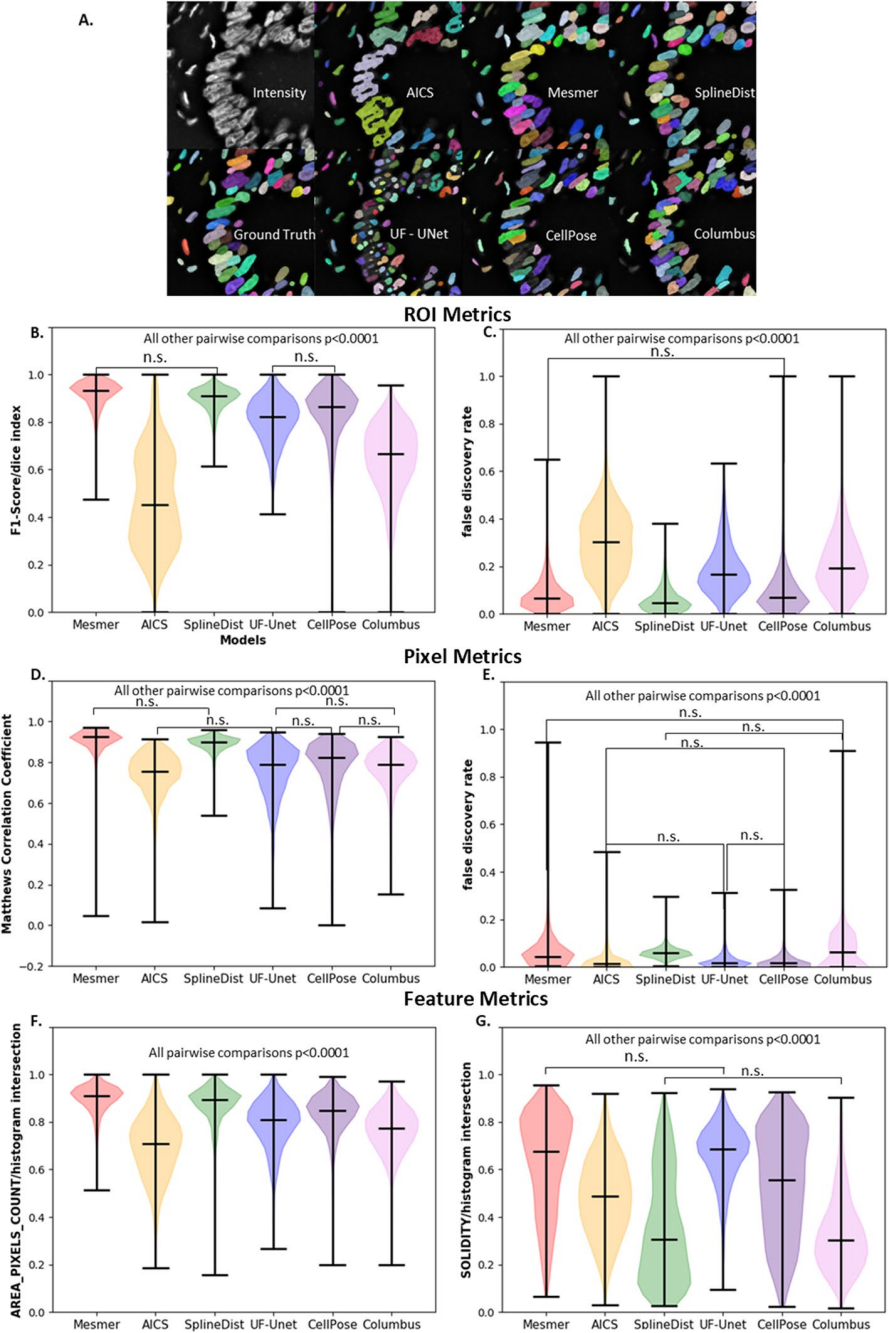
Nuclear Segmentation Labels and performance after fine-tuning UF-UNet and SplineDist. **A** Nuclear Segmentation Labels. ROI Level metrics for Nuclear Segmentation Labels for **B** F1 Score and **C** False Discovery Rate. Pixel Level metrics for **D** Matthews Correlation Coefficient and **E** False Discovery Rate. Feature Level metrics for Histogram Intersection of **F** Area and **G** Solidity. n.s.—not significant

Next, we compared segmentation methods after fine-tuning. The general expectation was that fine-tuning models should improve in segmentation metrics, and optimistically they would perform as well as Mesmer after fine-tuning. Figure 7A shows the segmentation results after fine tuning UF-UNet and SplineDist compared to all other segmentation pipelines. A detailed comparison of performance metrics for UF-UNet and SplineDist are shown in Table 5 to provide a closer look at differences between two fine-tuned models across all metric types. Table 5 and Fig. 7 highlight that the tools developed in this work are able to distinguish between high performing models, both before and after fine-tuning on target data, which is a common task in AI/ML data analysis.

**Table 5:** Mean ± std for nuclear segmentation evaluation metrics using trained SplineDist and UF-UNet models

Metric	Type	Pre-training		Post-training	
		SplineDist	UF-UNet	SplineDist	UF-UNet
*Region based measurements*					
F1 score	ROI	0.81 ± 0.09	0.16 ± 0.20	**0.89 ± 0.05**	0.80 ± 0.09
IoU score	ROI	0.69 ± 0.12	0.11 ± 0.17	**0.81 ± 0.07**	0.68 ± 0.13
False discovery rate	ROI	0.14 ± 0.09	0.72 ± 0.21	**0.06 ± 0.05**	0.18 ± 0.10
Fowlkes–Mallows index	ROI	0.81 ± 0.09	0.18 ± 0.19	**0.89 ± 0.05**	0.80 ± 0.09
*Pixel based measurements*					
IoU score	Pixel	0.72 ± 0.11	0.45 ± 0.20	**0.87 ± 0.03**	0.69 ± 0.13
Cohen’s kappa index	Pixel	0.09 ± 0.08	0.39 ± 0.18	**0.88 ± 0.04**	0.72 ± 0.16
Matthews correlation coefficient	Pixel	0.75 ± 0.12	0.37 ± 0.25	**0.89 ± 0.04**	0.75 ± 0.13
False discovery rate	Pixel	0.76 ± 0.10	0.39 ± 0.25	0.06 ± 0.02	**0.02 ± 0.03**
*Feature based measurements*					
Intersection area	Feature	0.82 ± 0.07	0.37 ± 0.22	**0.87 ± 0.07**	0.79 ± 0.10
Intersection perimeter	Feature	0.77 ± 0.10	0.32 ± 0.20	**0.82 ± 0.09**	0.73 ± 0.11
Intersection mean intensity	Feature	0.82 ± 0.08	0.52 ± 0.16	**0.89 ± 0.05**	0.80 ± 0.08
Intersection solidity	Feature	0.30 ± 0.20	0.24 ± 0.17	0.35 ± 0.22	**0.66 ± 0.12**

Bold indicates the top performing model for each metric shown

It can be seen from Table 5 and Fig. 7 that the metrics for UF-UNet significantly improved with training across all measured metrics. Figure 7B shows the ROI level scores between different models after training SplineDist and UF-UNet. Of note is that after fine-tuning, UF-UNet was not significantly different from CellPose when F1 scores were compared (*p* = 0.6545) and had significantly lower false discovery rates (Table 5). Interestingly, the ROI false positive metric was the smallest for SplineDist after training even compared to Mesmer (Additional file 1: Fig. S145). The F1 score for SplineDist was also found to not be significantly different from Mesmer (*p* = 0.0022).

The PL scores from SplineDist and UF-UNet also significantly improved with training, as seen in Fig. 7D, E. The results from UF-UNet after training were not significantly different from Columbus and CellPose for MCC loss (*p* = 0.2726 and *p* = 0.9620 respectively, Fig. 7D). The improvement in PL scores from UF-UNet can also be seen in Table 5, where the mean ± std for MCC for pre-trained UF-UNet model changed from 0.37 ± 0.25 to 0.75 ± 0.13 after training. SplineDist also showed significant improvement in pixel level scores with its mean ± std for MCC changing from 0.75 ± 0.12 to 0.89 ± 0.04, showing no statistically significant difference between Mesmer and trained SplineDist. After training, the FDR for UF-UNET became comparable to that of AICS and CellPose (*p* = 0.997 and 0.6528, respectively). The changes in *p* value for SplineDist and UF-UNet, along with the changes in their evaluation metrics, highlight the improvement in their segmentations with training, with the results from SplineDist becoming comparable to those of Mesmer, and UF-UNet becoming comparable with CellPose and Columbus. These comparisons are important since it could be seen in “Segmentation comparison” section that Mesmer consistently exhibited statistically higher performance than other models before pre-training.

The improvement in scores can also be observed from the feature level metrics in Table 5 and can be seen in Additional file 1: Figs. S180–S208. While the score for histogram intersection of area (Fig. 7F) for UF-UNET and SplineDist improved with training as seen in Table 5, it was still significantly different from that of Mesmer. However, the histogram intersection of Solidity (Fig. 7G) for UF-UNET after training was found to not be significantly different from that of Mesmer (*p* = 0.0002).

### Metric selection using principal component analysis

**Fig. 8:**
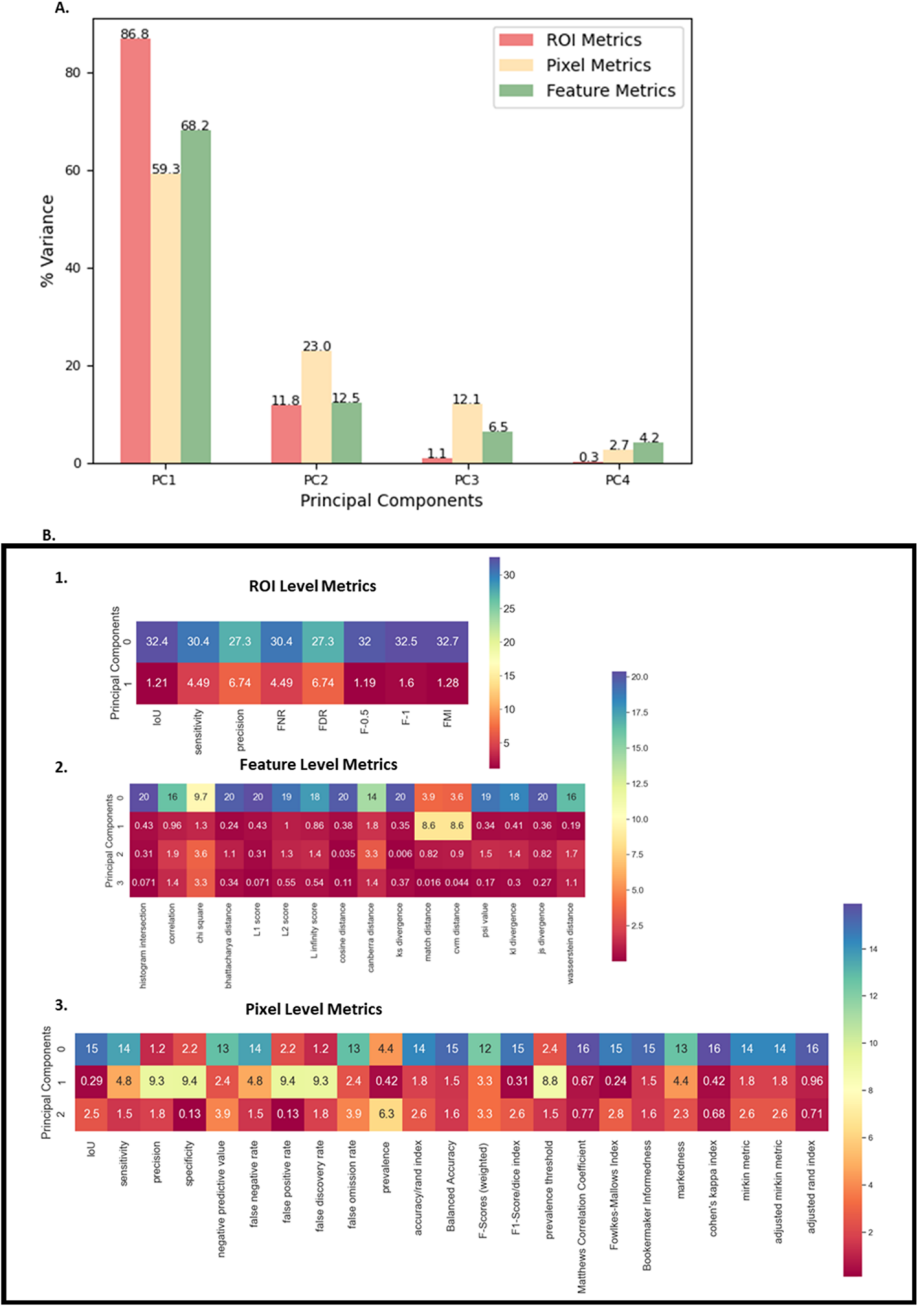
ROI based Metric Selection for Nuclear Segmentation for Pre-trained Models using PCA. **A** PCA variance percentage for all metrics. **B** Absolute coefficient values (eigenvectors) multiplied by variance percentage for components adding to 90% for (1) ROI Level Metrics (2) Feature Level Metrics and (3) Pixel Level Metrics

From the hundreds of possible Feature level metrics to the dozens of PL and ROI metrics, quickly and efficiently identifying metrics that best represent model performance is challenging. Two simple machine learning approaches were used to select a small number of relevant performance metrics for a dataset. Principal Component Analysis (PCA) was used for selecting metrics for the purpose of comparing models. To select ROI, PL, and feature level metrics, PCA was implemented on the ROI, Pixel, and feature metrics separately for nuclei and cytoplasm segmentation. The absolute coefficient values and percentage variance from PCA were multiplied to get the features with maximum variance and these were selected for model comparison. The 69 metrics identified in this manuscript were collated by performing an extensive literature review to incorporate all assessment metrics the community commonly uses. The libraries are easily extensible and open source for easy expansion to other metrics the community finds of interest in the future. Figure 8A shows the variance ratio percentage (explained variance ratio*100) for all the components and Fig. 8B shows the heatmap for absolute coefficient values (eigenvectors) multiplied by their variance percentage from PCA for pre-trained models for nuclear segmentation. For ROI metrics, the first two components were found to account for 98.5% data variance (Principal component 1 (PC1) 86.8% and PC2 11.7%); for PL metrics, the first 3 components were found to explain more than 90% of the data variance and for feature level metrics, the first 4 components explained more than 90% of the data variance. Figure 8A shows a bar plot of each of these values. The remaining PCA plots for nuclear and cytoplasm segmentation metrics have been included in Additional file 1: Figs. S209–S214. A Catboost Classifier model was also implemented for PL metrics of Cytoplasm segmentations to provide an example of another method for metric selection. The feature importance table from the Catboost model has been included in Additional file 1: Fig. S213. The results from PCA and Catboost classifier were similar as can be seen in Additional file 1: Fig. S213. Catboost was not used for the main feature selection in this paper but was used as a secondary method to validate that the features from the PCA were robust to feature selection methods. We used Catboost as an example of a more modern method that yields nearly identical results.

## Discussion

Selecting evaluation metrics for evaluating segmentation quality is essential to the process of selecting a good method for a given dataset. We have demonstrated the advantages of having access to a pipeline that generates a comprehensive set of metrics and the methods to select them. While the trend has been to use a few metrics [14, 15]**,** our proposed methods enable researchers to easily obtain an array of evaluation metrics, identify metrics that differentiate approaches, and select metrics of scientific importance or relevance. We show that these tools can be applied to traditional segmentation pipelines, novel deep learning-based pipelines, as well as in the context of comparing between diverse segmentation approaches, within a model as a function of training (either on data or epoch), and also between highly performing fine-tuned models. The results in this paper highlight the importance of having access to metrics at ROI, pixel, and feature level, with each method providing essential and distinctive information regarding segmentation quality.

The results for nuclear and cytoplasm segmentation using models that had not been fine-tuned on TissueNet validated each metric, since Mesmer was expected to outperform all other segmentation pipelines and did according to the majority of the metrics [18]. While it is not surprising that Mesmer outperforms the models that were not trained on the same TissueNet dataset, the reasons for this performance compared to other methods that are fine-tuned on TissueNet are beyond the scope of this paper to evaluate as the focus of this paper is to extract the metrics and not understand the reason behind the performance of imaging algorithms. The ROI metrics in Table 2 and Fig. 3A-H highlighted the quality of region-based segmentation. The importance of PL metrics to quantify foreground versus background segmentation could be observed through metrics like MCC in Table 3 and Fig. 4A–H. As MCC depends on both the positive and negative classes, it can be a better indicator of the quality of segmentation in case of binary classification, compared to metrics like F1 score and accuracy [30, 31]. The extraction of features like cell area, perimeter, mean intensity, and solidity also helped in deducing the quality of segmentation, providing more fine-grained insights into what types of morphological errors a particular algorithm made. A list of distance metrics like histogram intersection, L1 score, and JS divergence were used to compare the ground truth and predicted feature metrics. The results from evaluation metrics for nuclear segmentation using pre-trained models proved the significantly better performance of models like Mesmer, SplineDist and CellPose over segmentations from AICS, UF-UNet and Columbus for most metrics. The performance (ROI, PL, or feature level) from Columbus and CellPose for cytoplasm segmentation was better than AICS but worse than Mesmer as seen in Figs. 3E–H, 4E–H and 5E–H. This was expected since the pipeline used to segment using AICS was not originally meant for cytoplasm, but curvilinear structures like Sec61 beta and Lamin B1 [20]**.** Results from ROI, PL and feature level metrics provided a more detailed picture of how and what type of errors each segmentation approach was showing.

UF-UNet and SplineDist models were also trained on the TissueNet dataset to obtain comparable results as Mesmer for nuclear segmentation. The results from training UF-UNet on TissueNet over different iterations and dataset sizes were included in “Metric assessment as a function of network training for nuclear segmentation” section for the purpose of highlighting the change in metrics with varying training parameters. The discrepancies between ROI, PL and feature based metrics for training UF-UNet with varying training sizes in Fig. 6 highlighted the importance of obtaining the distinct types of metrics. While fine-tuning UF-UNet on all training data improved the ROI based scores, it also made the pixel level scores worse, likely because of a decrease in the number of pixel level true positives and an increase in false negatives and false positives (Additional file 1: Figs. S128, S130 and S131, respectively). According to Falk et al. [17], UF-UNet should only require approximately 10 images for training in cases of extremely complex datasets, since TissueNet comprises of 6 different tissue types and imaging platforms, we started training with 15 images, and saw significant improvements, but more training data showed significant improvements across ROI, Pixel, and Feature metrics. The evaluation scores for UF-UNet further improved with more training; however, it was a trade-off between training time and quality of segmentation. All ROI, pixel, and feature level metrics for SplineDist and UF-UNet were seen to improve with training as seen in 4.3. Through a combination of hypothesis testing and evaluation metrics, we could prove that UF-UNet and SplineDist improved with training on TissueNet for nuclear segmentation.

Principal Component Analysis was used for extraction of metrics that best separate models in a dimensionally reduced space from the extensive list of ROI and pixel level metrics. More modern classification methods, Catboost Classifier [32], were also utilized to identify metrics that best correlated with model performance. Similar results were found for both approaches as seen in Fig. 8 and Additional file 1: Fig. S213. For feature level metrics, it is recommended to perform correlation analysis on the features or spot checking, followed by dimensionality reduction on the Histogram metrics. Researchers can also select features based on their pertinence to research, like cell morphology vs intensity. Correlation analysis can also be used to shortlist ROI and pixel level metrics [22, 24, 33], but this type of feature selection is hypothesis driven and cannot be generalized, unlike the methodology outlined here. Common community standards in the field of feature assessment do not currently exist. However, recent publications [34, 35] have shown that the community is aware of this problem and that a large effort is underway to develop these standards. Producing these artifacts is therefore seen as outside of the scope of this particular paper but an area of active research for the Authors. Whenever these standards are developed/released the Authors will incorporate these standards into the unit tests for each feature.

## Conclusions

The purpose of our work is to share the vast variety of evaluation metrics, implemented in three tools and a single pipeline. These tools are fully open source, scalable to any image size, available as containers, in GUI form in the Web Image Processing Pipeline platform, as Common Workflow Language tools, and from the command line. These metrics, as we have demonstrated through our results, are important for a qualitative and quantitative comparison of imaging algorithms. Our pipeline enables researchers to analyze their segmentations using a wide range of ROI, pixel, and feature level metrics efficiently and comprehensively across all common use cases in both traditional and machine learning workflows. Through a range of results, we have proven the importance of obtaining ROI level metrics to analyze segmentations at an instance level, pixel level metrics to analyze foreground and background segmentations, and feature level metrics to understand the properties of segmented regions like cell morphology.

### Supplementary Information

**Additional file 1:** 207 region, pixel, and feature comparison graphs, PCA and CatBoost feature importance metrics, across both cell nuclei and cytoplasm.

**Additional file 2:** Formula for all extracted metrics.

**Additional file 3:** Supplemenatary Tables 1–7. Tools and their github/dockerhub locations, and AI model performance as a function of sample size and training epochs.

## Data Availability

The datasets used and generated during the current study are available from the corresponding author on reasonable request.
