# Supplementary material for: Unbiased image segmentation assessment toolkit for quantitative differentiation of state-of-the-art algorithms and pipelines
Source: BMC Bioinformatics. 2023 Oct 12;24:388. doi: 10.1186/s12859-023-05486-8 (PMC10568754; doi:10.1186/s12859-023-05486-8)

Supplementary Figures

Pre-Trained Models—ROI Level Metrics. Figures 1-9: Nuclear Segmentation. Figures 10-18: Cytoplasm Segmentation

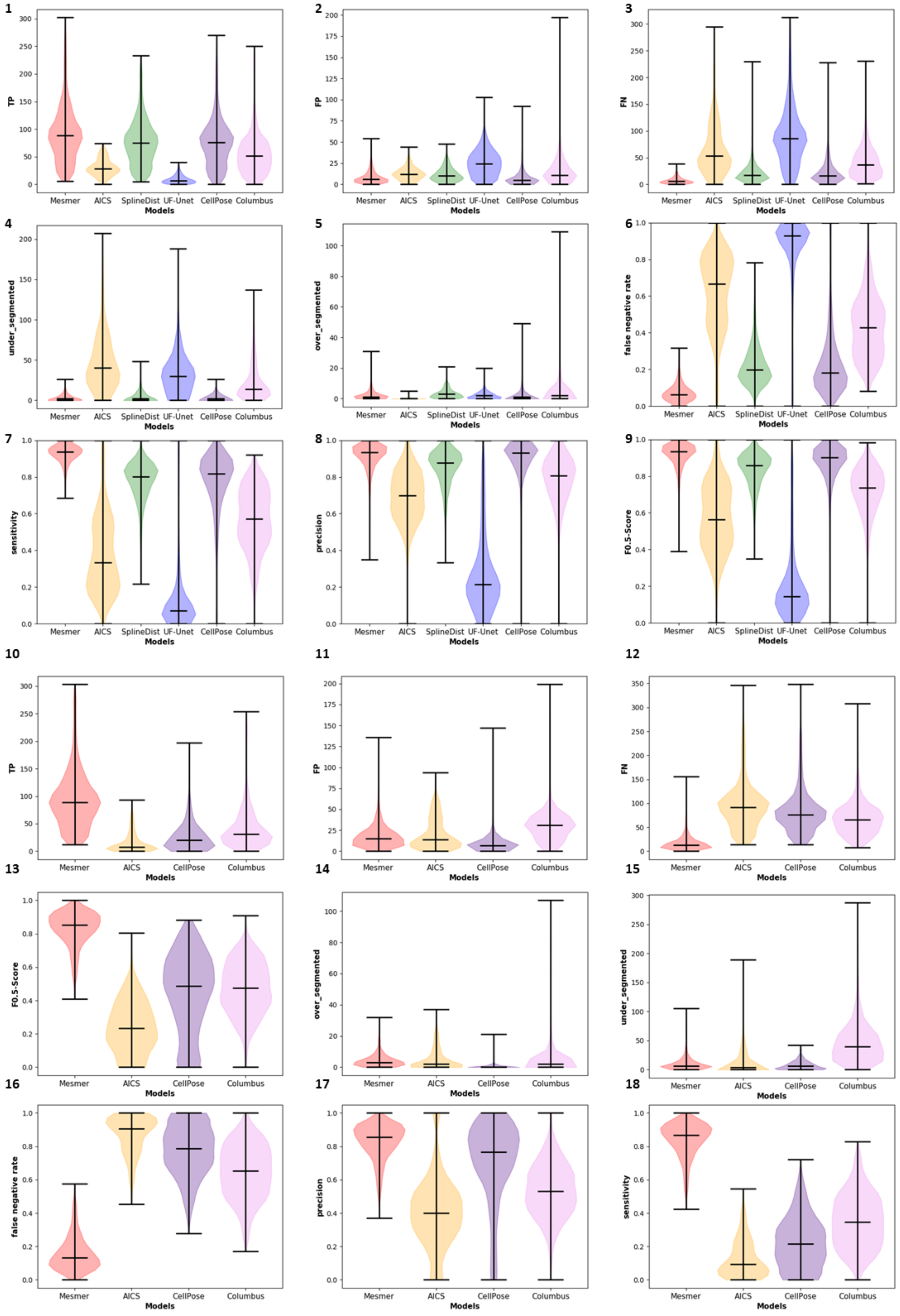

Pre-Trained Models—Pixel Level Metrics. Figures 19-41: Nuclear Segmentation. Figures 42-64: Cytoplasm Segmentation

19

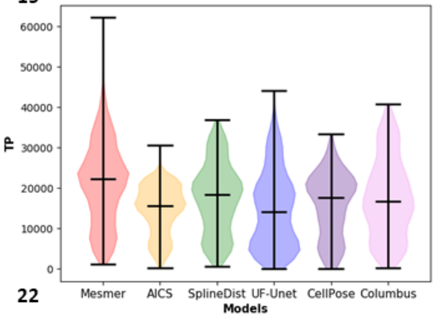

20

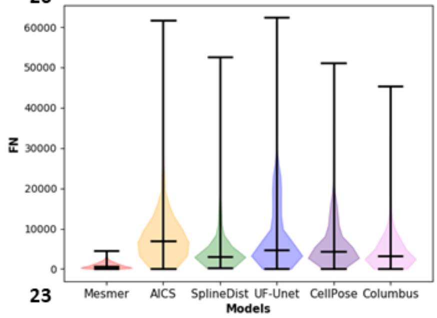

21

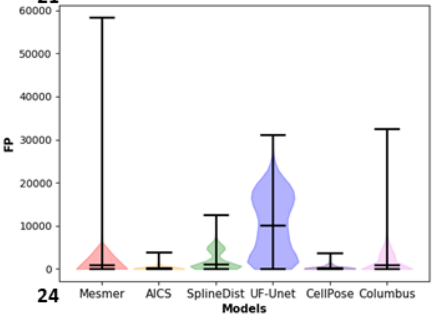

22

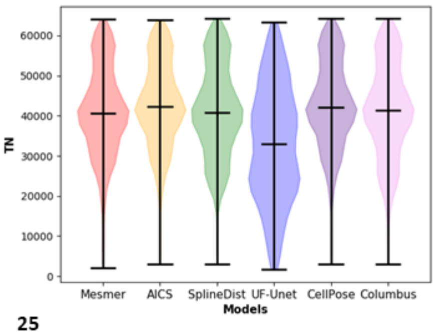

23

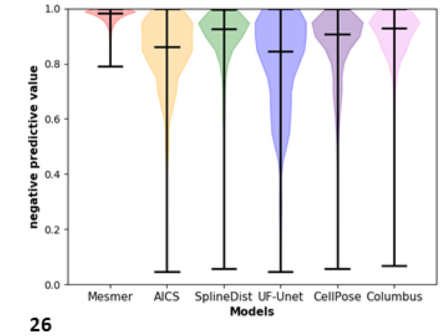

24

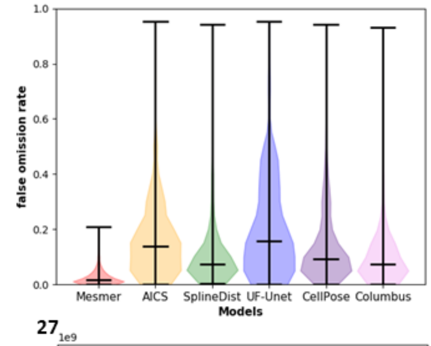

25

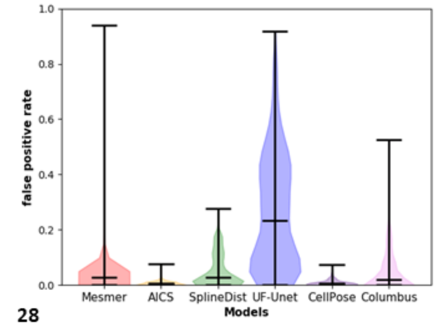

26

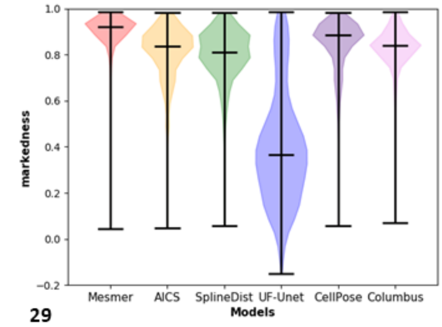

27

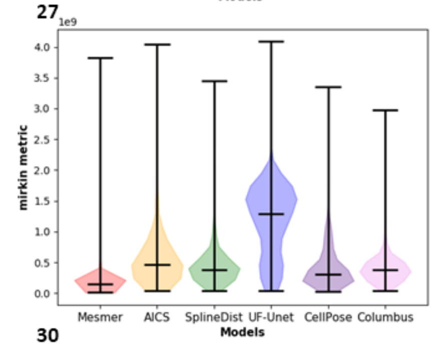

28

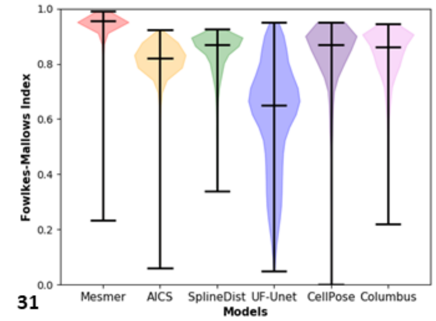

29

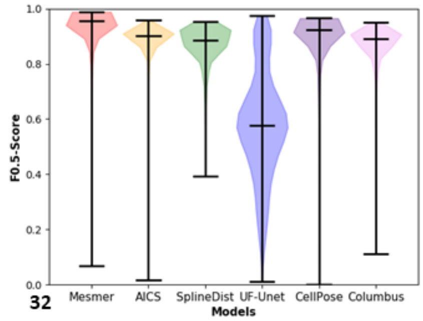

30

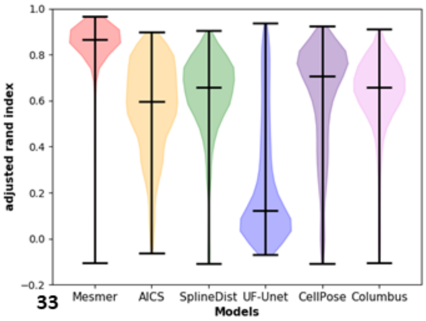

31

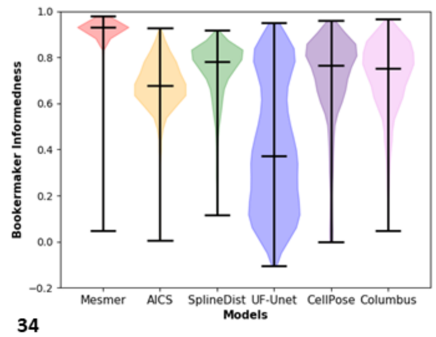

32

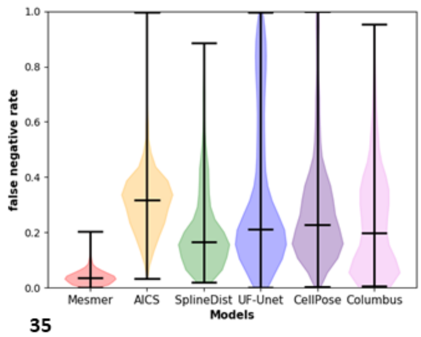

33

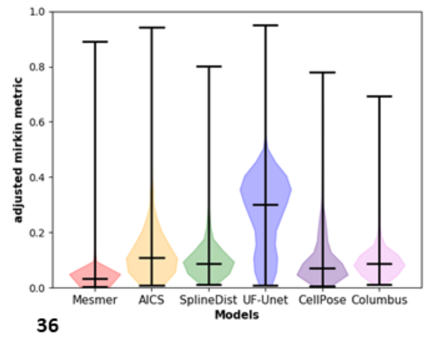

34

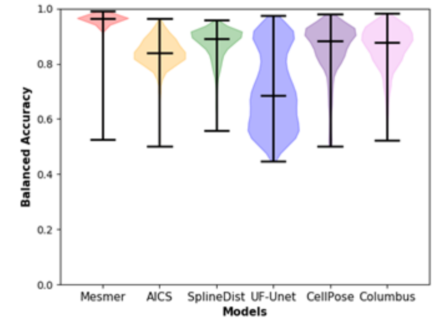

35

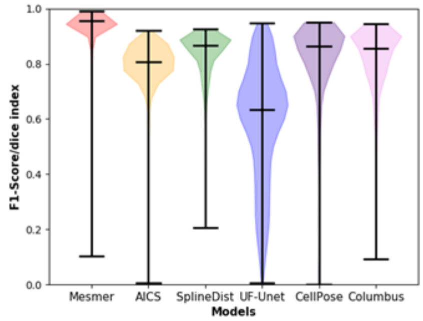

36

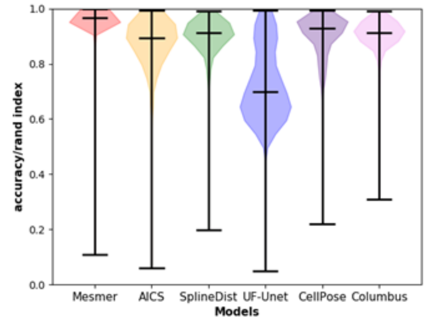

37
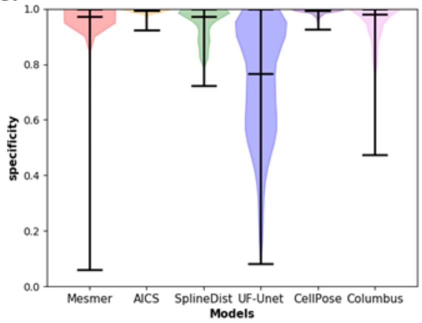

38
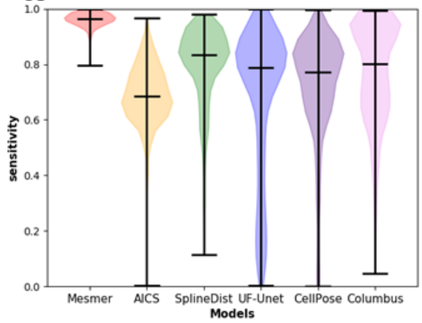

39
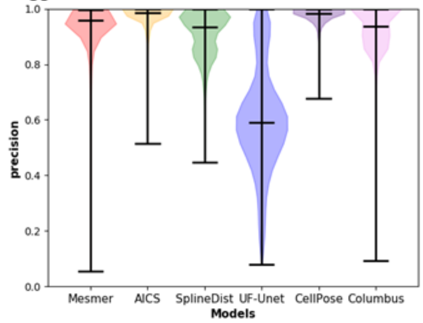

40
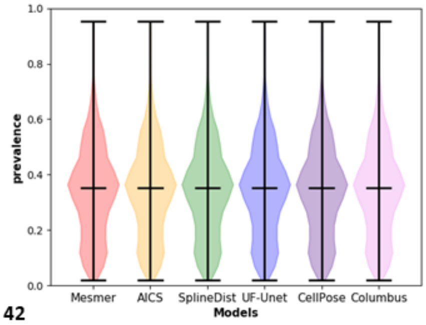

41
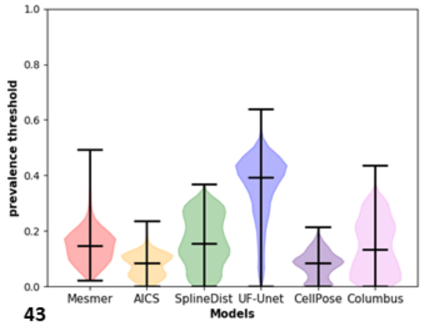

42
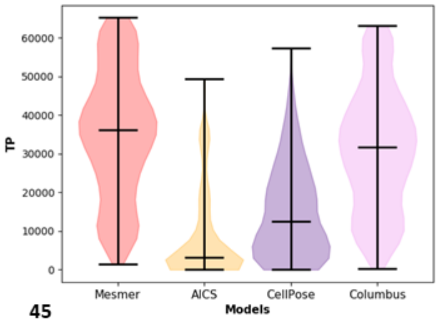

43
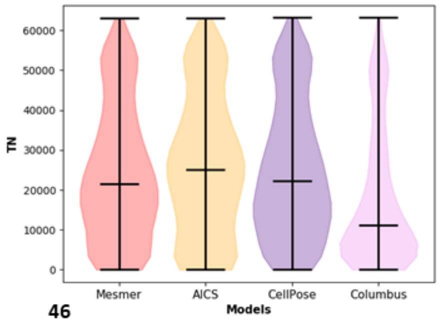

44
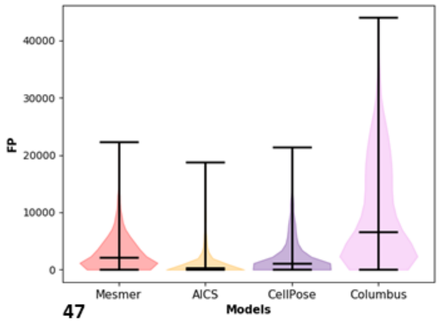

45
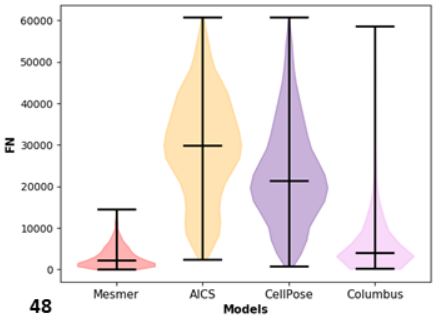

46
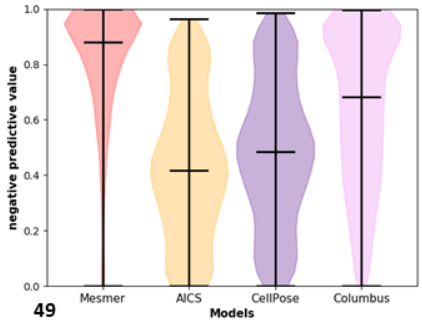

47
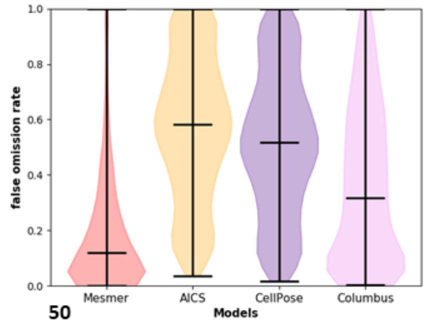

48
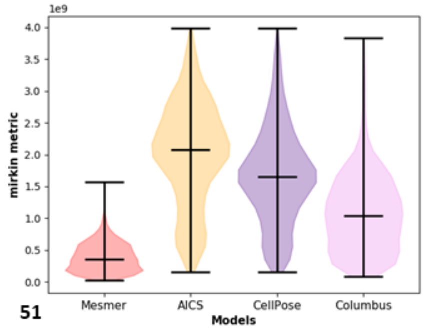

49
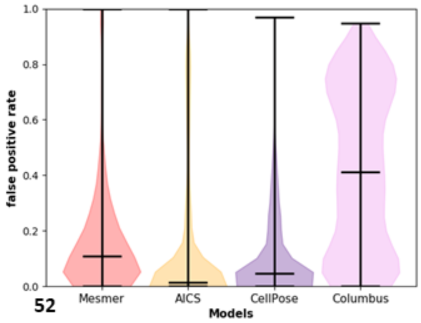

50
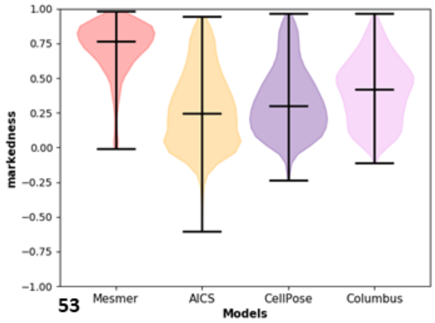

51
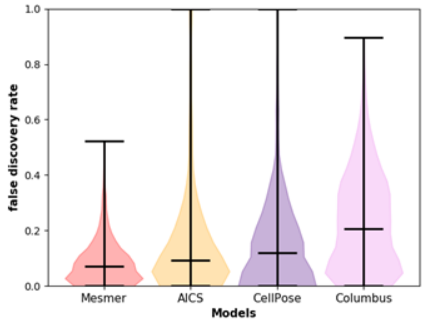

52
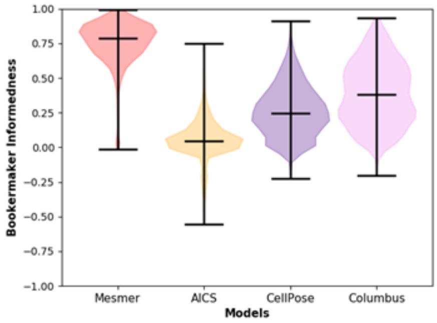

53
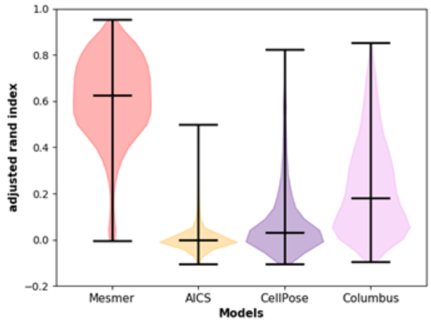

54

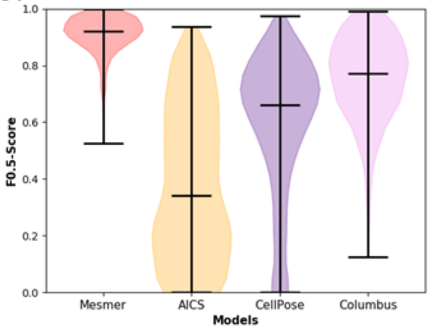

55

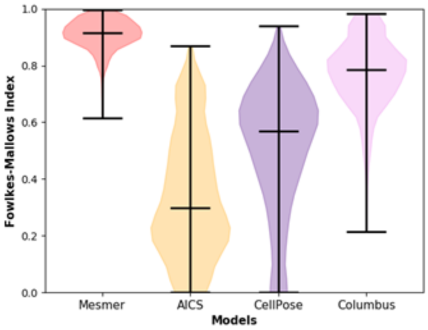

56

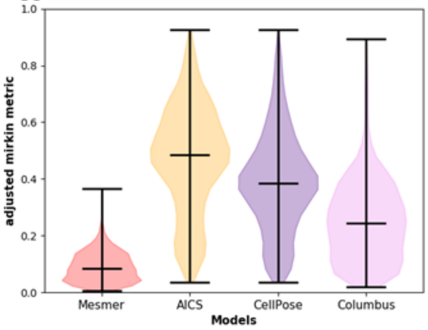

57

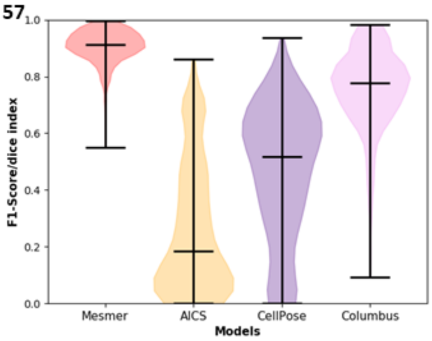

58

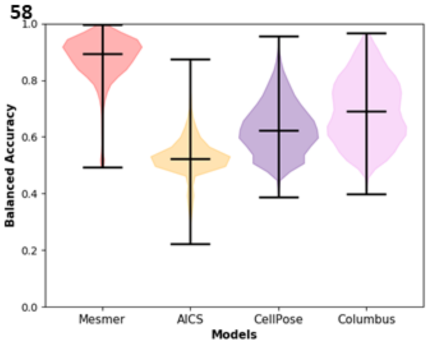

59

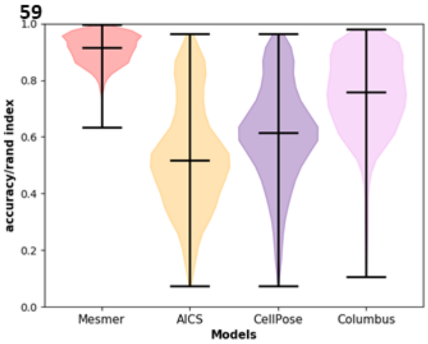

60

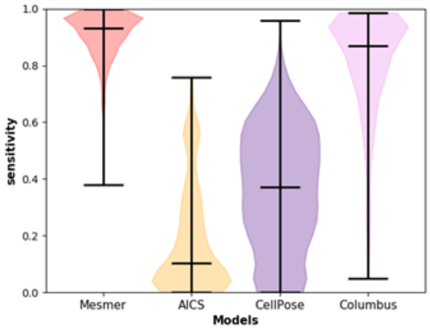

61

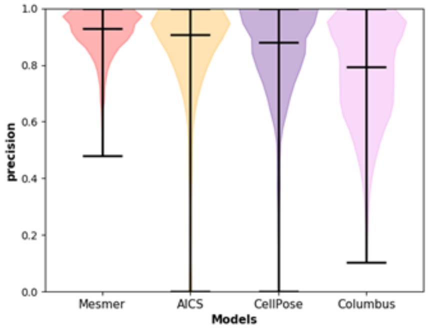

62

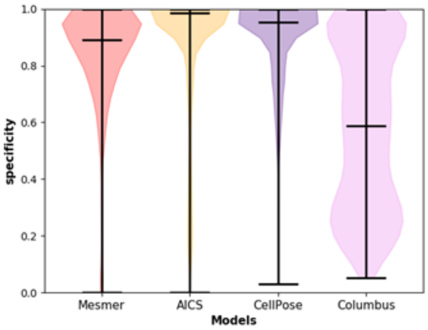

63

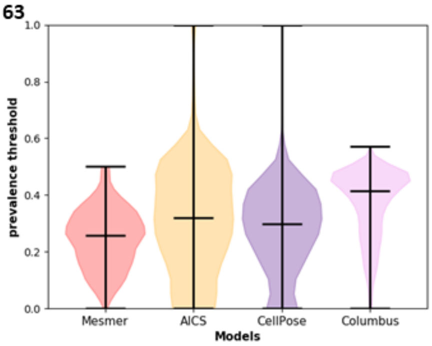

64

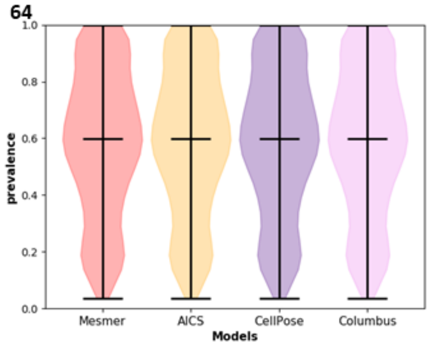

Pre-Trained Models—Feature Level Metrics. Figures 65-91: Nuclear Segmentation. Figures 92-118: Cytoplasm Segmentation

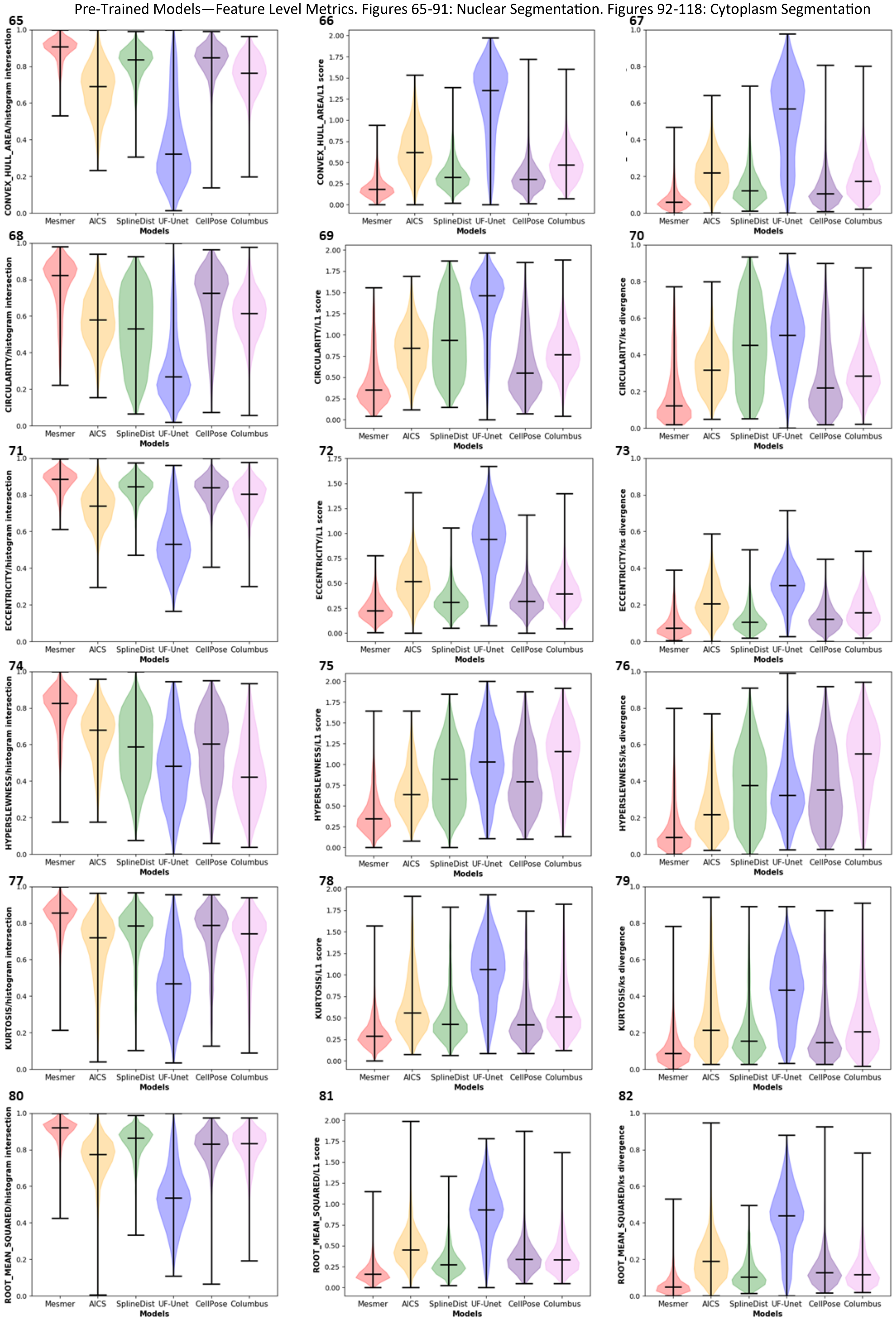

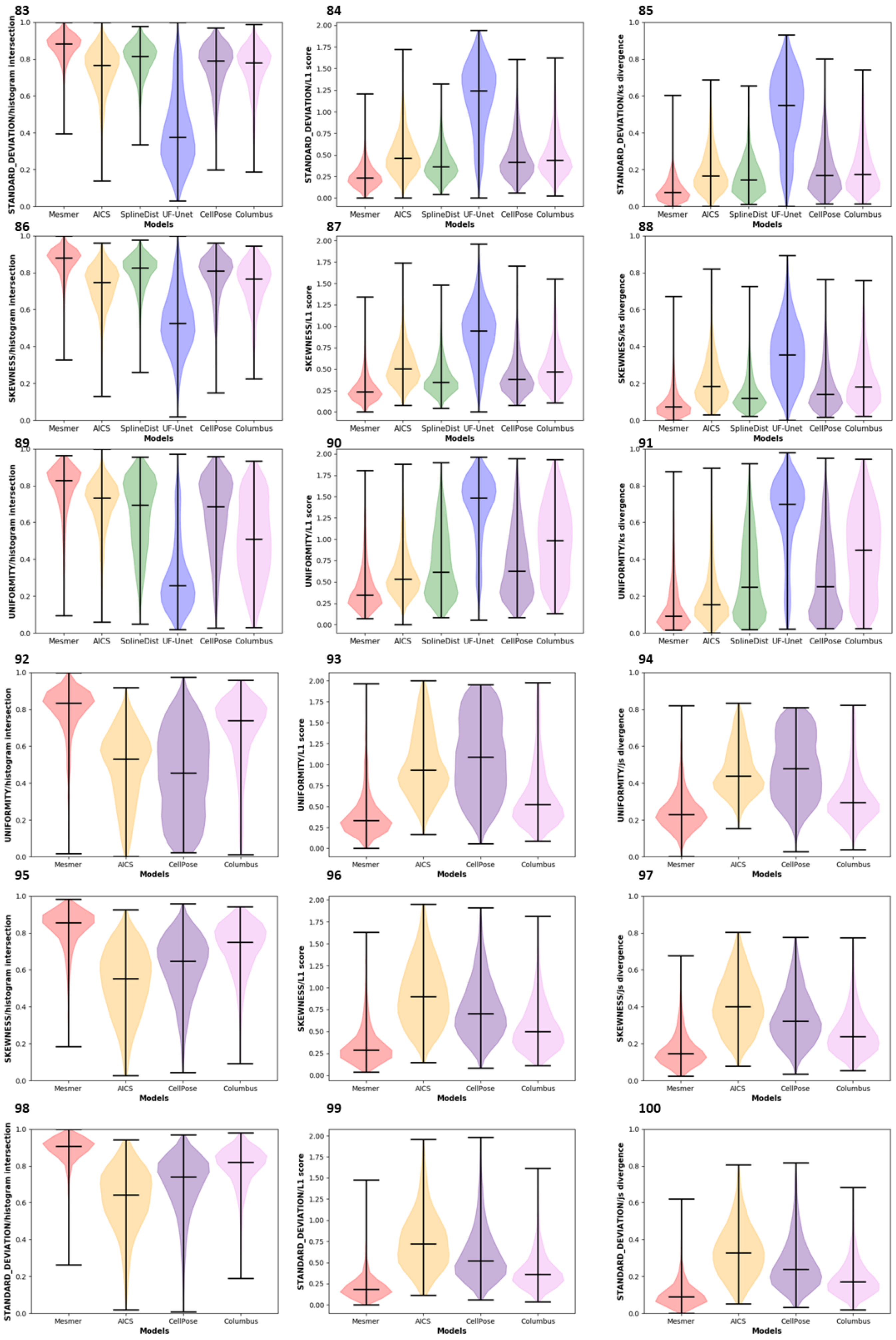

83
STANDARD_DEVIATION/histogram intersection
84
STANDARD_DEVIATION/L1 score
85
STANDARD_DEVIATION/ks divergence
86
SKEWNESS/histogram intersection
87
SKEWNESS/L1 score
88
SKEWNESS/ks divergence
89
UNIFORMITY/histogram intersection
90
UNIFORMITY/L1 score
91
UNIFORMITY/ks divergence
Mesmer AICS SplineDist UF-Unet CellPose Columbus
92
UNIFORMITY/histogram intersection
93
UNIFORMITY/L1 score
94
UNIFORMITY/js divergence
95
SKEWNESS/histogram intersection
96
SKEWNESS/L1 score
97
SKEWNESS/js divergence
98
STANDARD_DEVIATION/histogram intersection
99
STANDARD_DEVIATION/L1 score
100
STANDARD_DEVIATION/js divergence
Mesmer AICS CellPose Columbus
Models

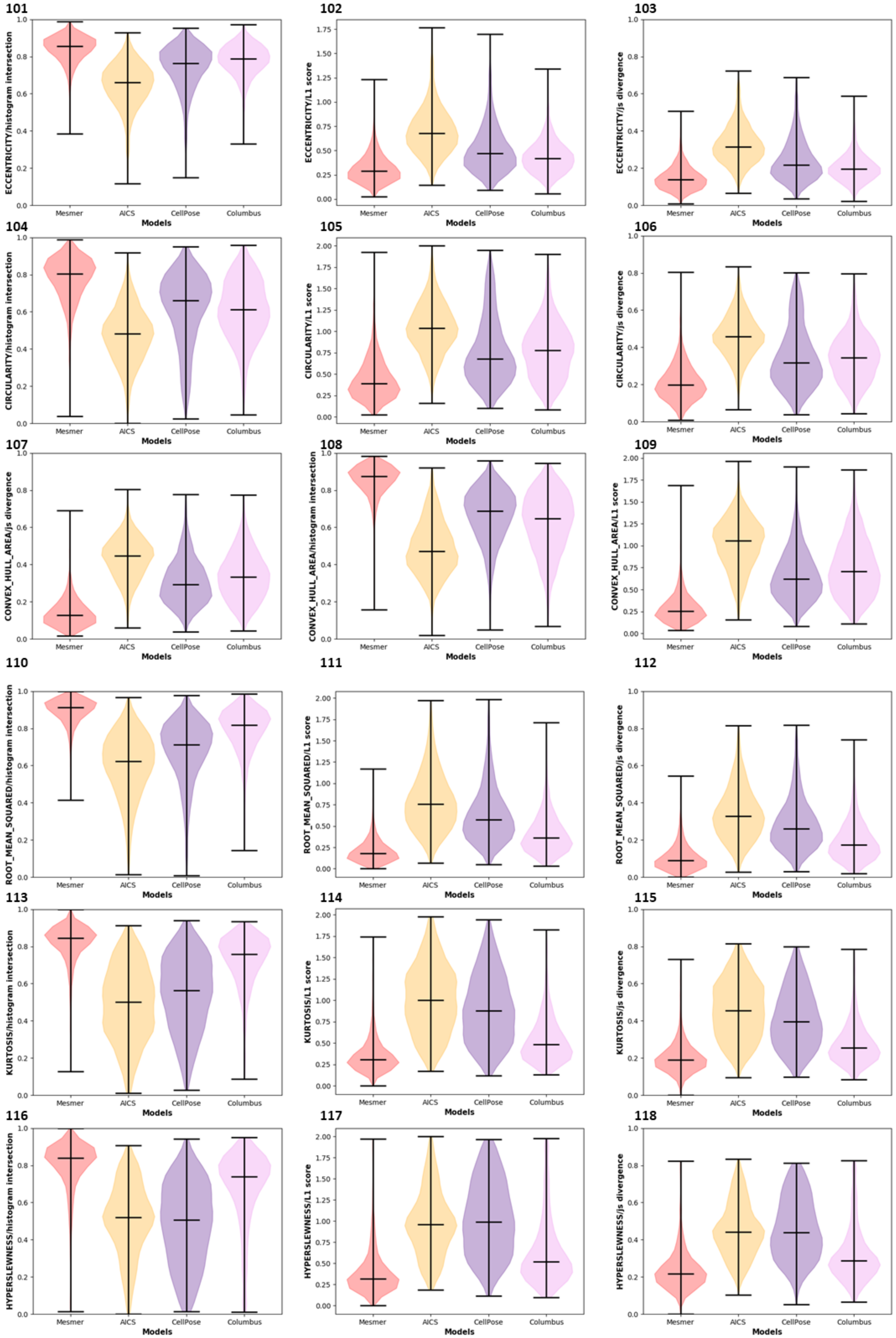
101
ECCENTRICITY/histogram intersection
102
ECCENTRICITY/L1 score
103
ECCENTRICITY/js divergence
104
CIRCULARITY/histogram intersection
105
CIRCULARITY/L1 score
106
CIRCULARITY/js divergence
107
CONVEX_HULL_AREA/js divergence
108
CONVEX_HULL_AREA/histogram intersection
109
CONVEX_HULL_AREA/L1 score
110
ROOT_MEAN_SQUARED/histogram intersection
111
ROOT_MEAN_SQUARED/L1 score
112
ROOT_MEAN_SQUARED/js divergence
113
KURTOSIS/histogram intersection
114
KURTOSIS/L1 score
115
KURTOSIS/js divergence
116
HYPERSLEWNESS/histogram intersection
117
HYPERSLEWNESS/L1 score
118
HYPERSLEWNESS/js divergence
Mesmer
AICS
CellPose
Columbus
Models

UNet Training for Nuclear Segmentation. Figures 119– 127 ROI Metrics. Figures 128—137 Pixel Metrics. Figures 138— 143 Feature Metrics.

119

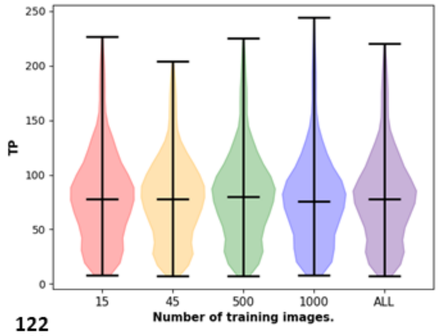

120

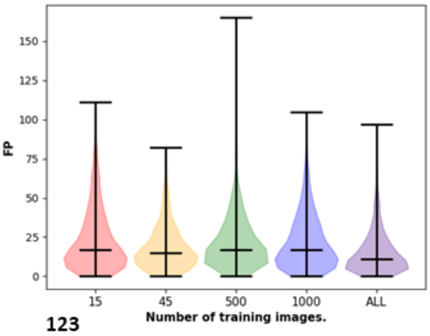

121

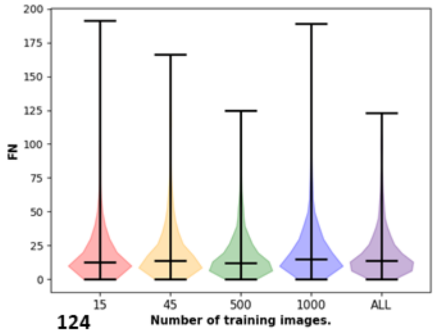

122

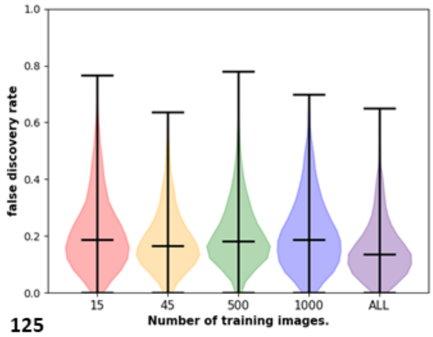

123

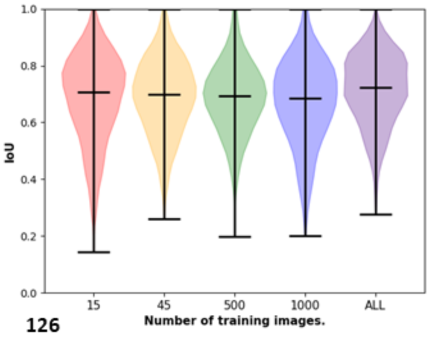

124

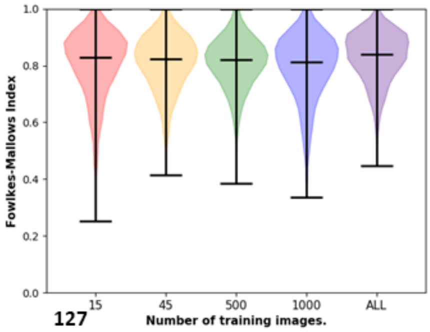

125

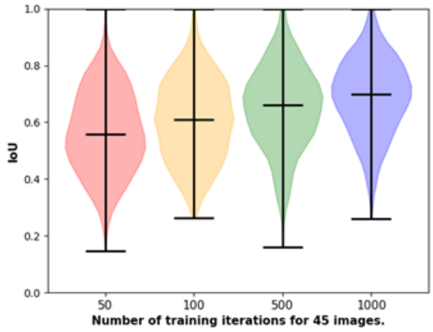

126

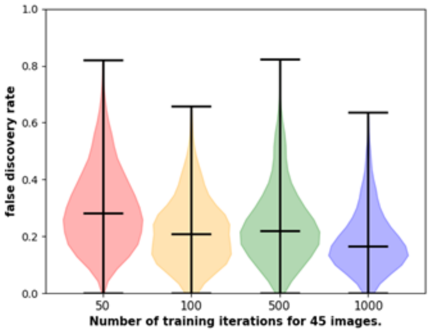

127

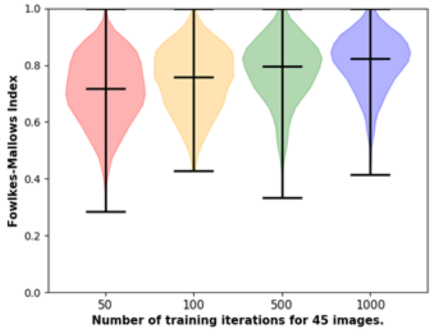

128

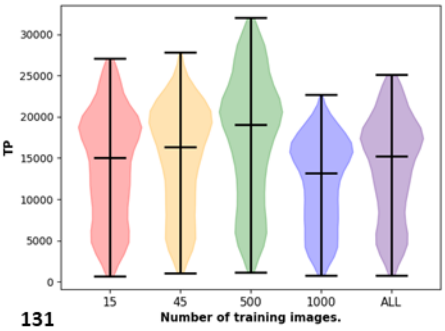

129

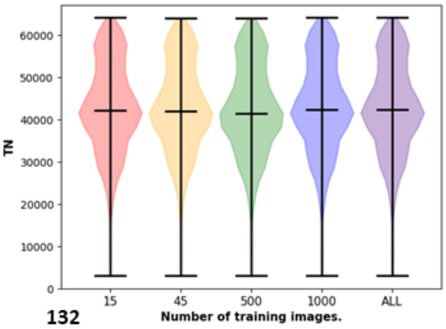

130

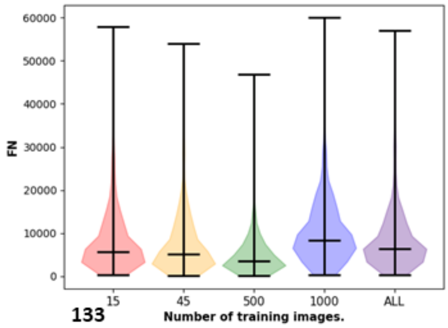

131

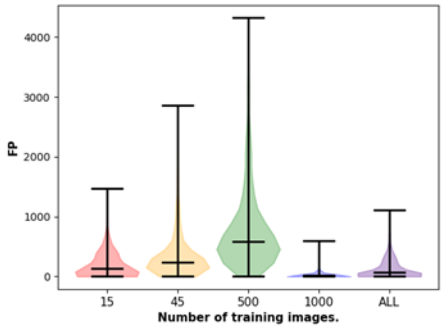

132

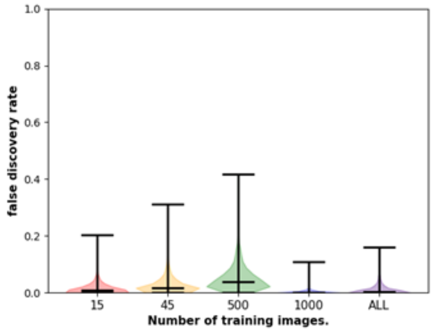

133

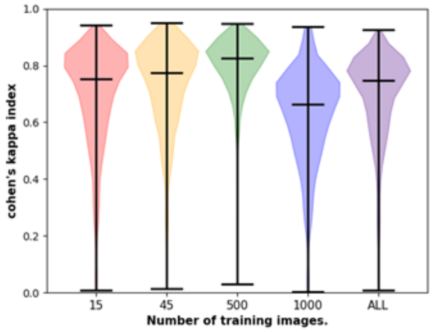

134

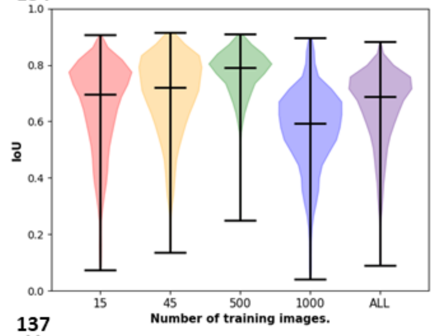

135

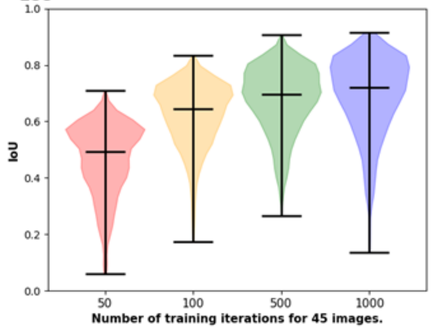

136

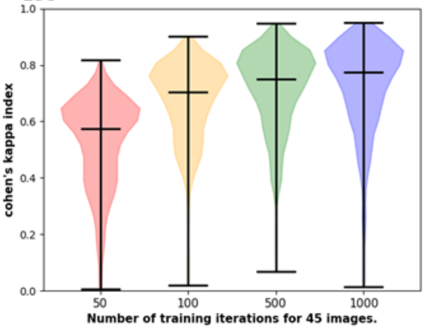

137

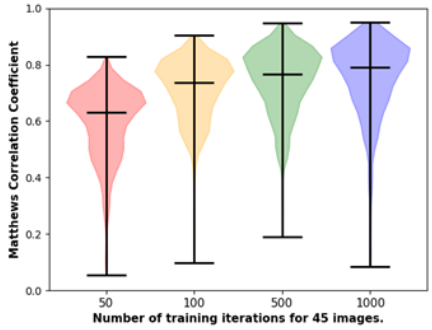

138

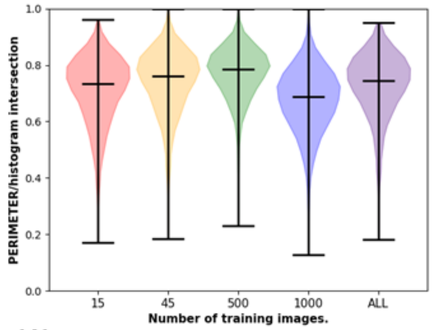

139

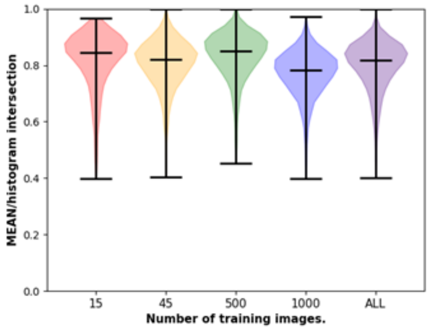

140

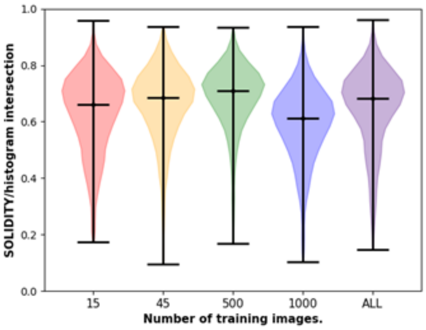

141

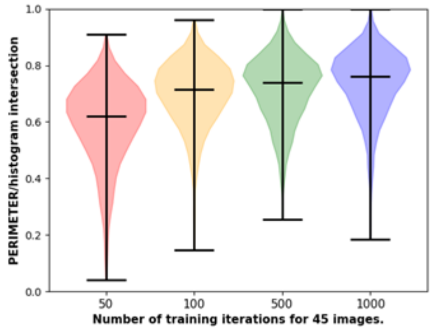

142

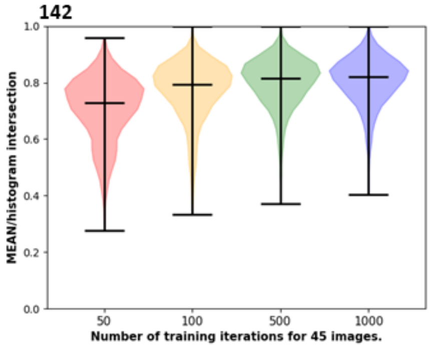

143

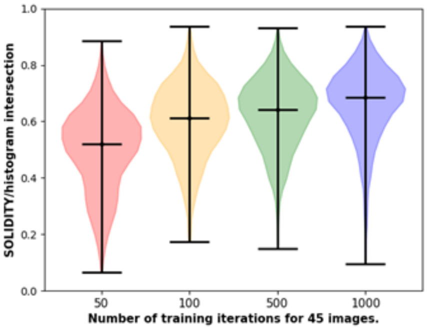

Trained Models for Nuclear Segmentation. Figures 144— 154 ROI Metrics. Figures 155— 179 Pixel Metrics. Figures 180— 208 Feature Metrics.

144

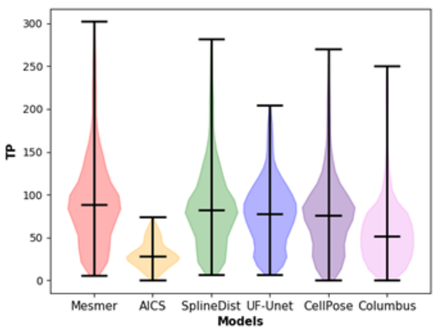

145

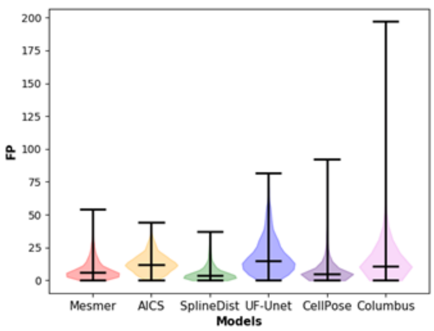

146

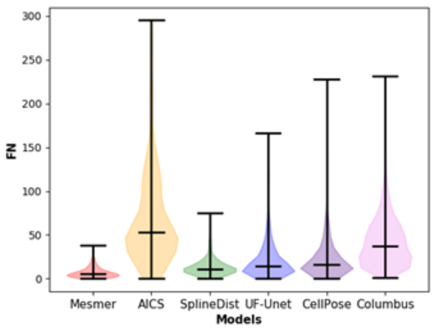

147

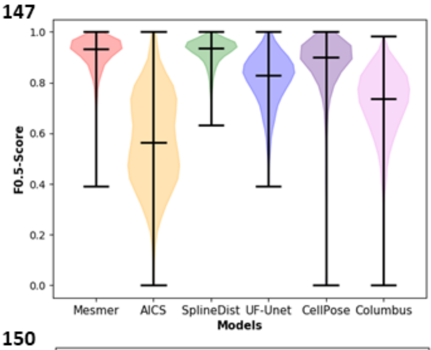

148

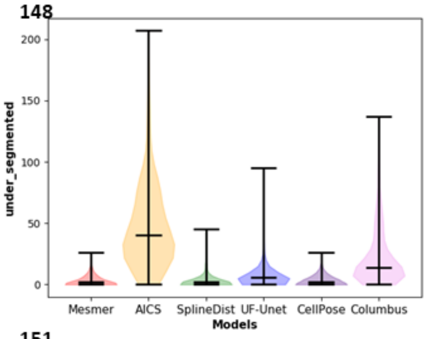

149

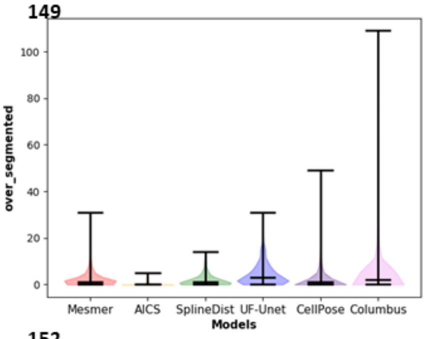

150

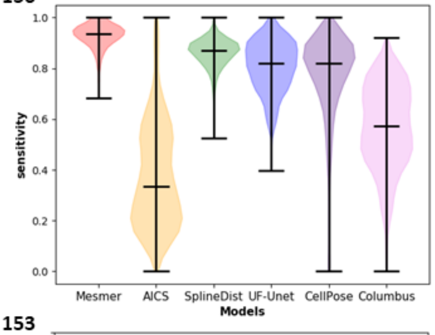

151

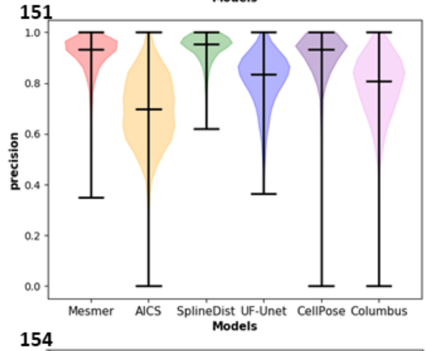

152

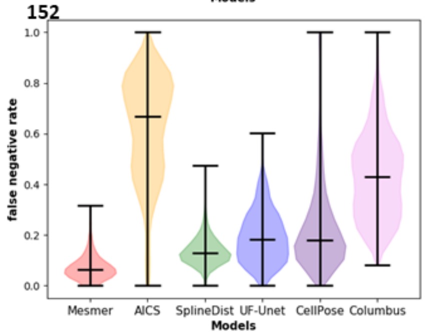

153

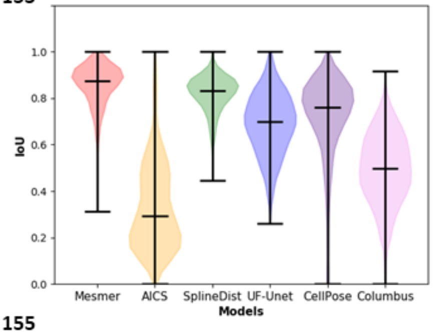

154

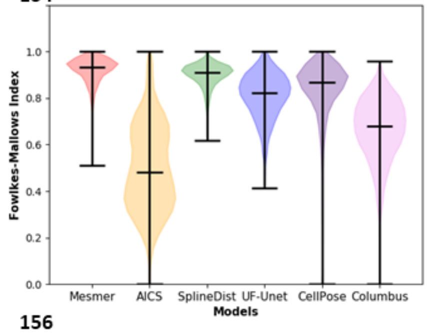

155

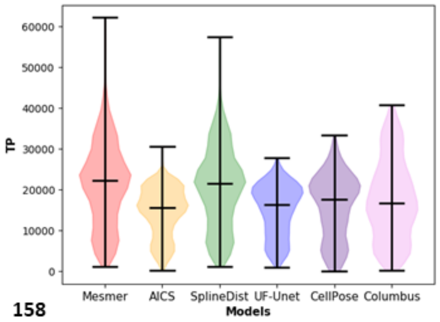

156

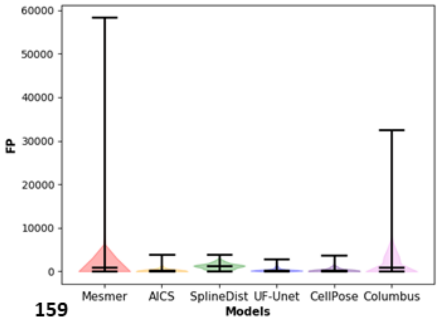

157

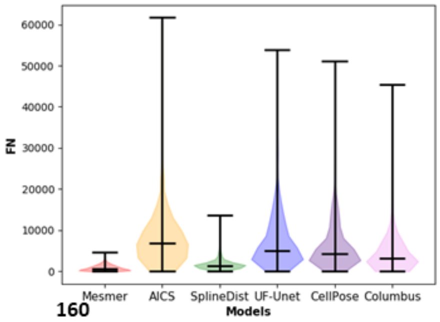

158

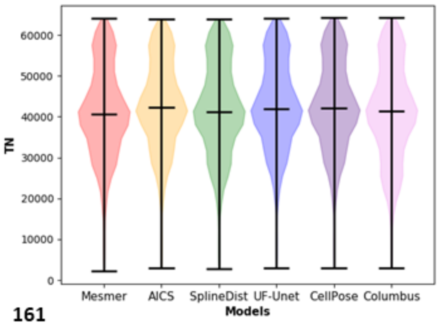

159

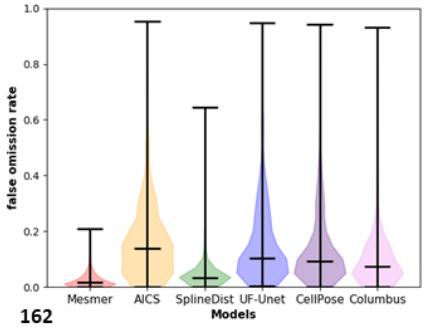

160

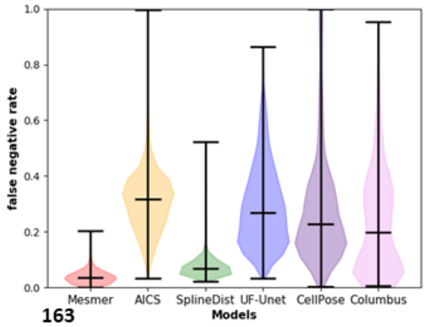

161

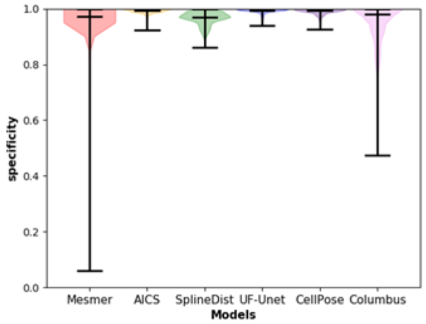

162

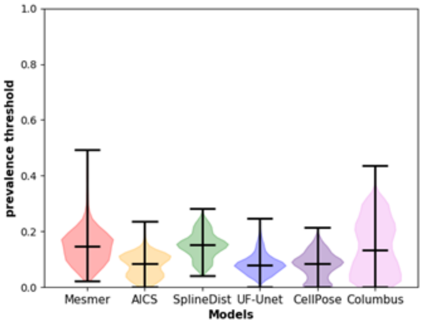

163

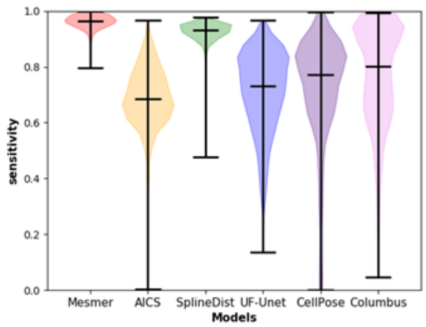

164

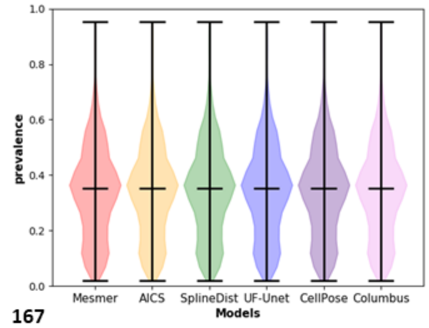

165

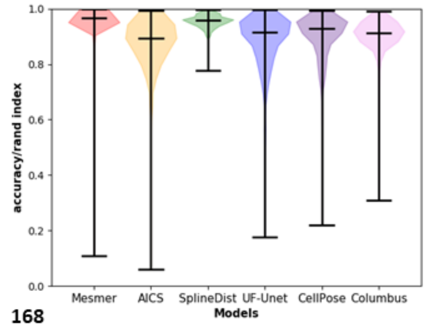

166

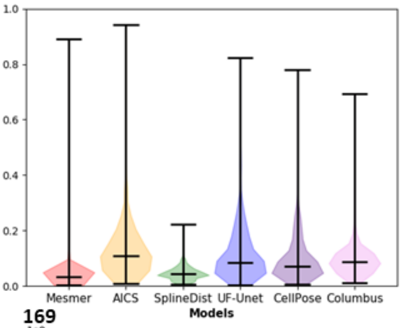

167

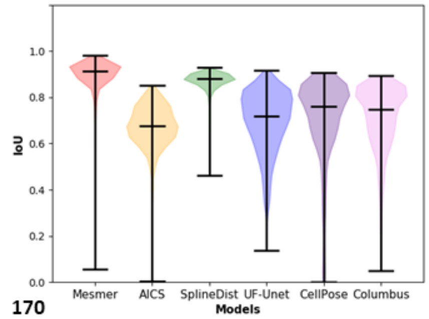

168

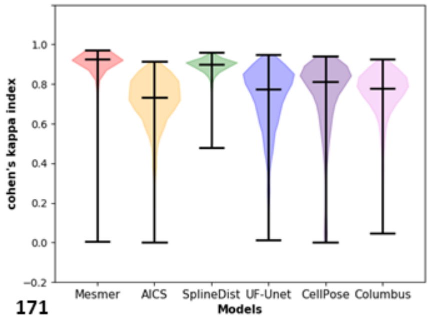

169

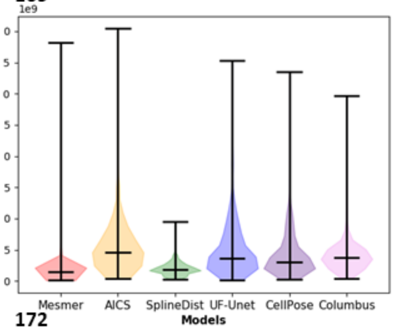

170

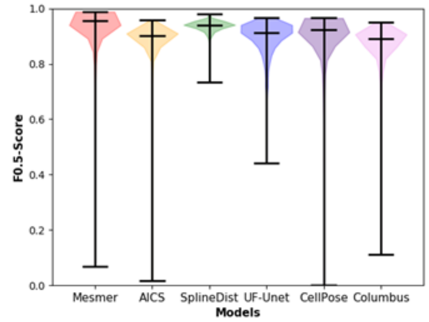

171

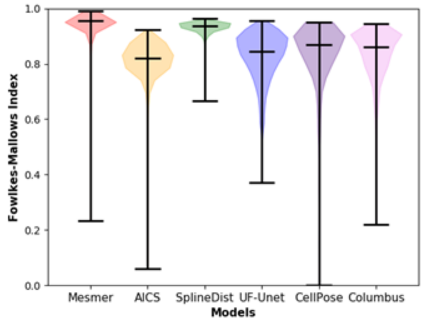

172

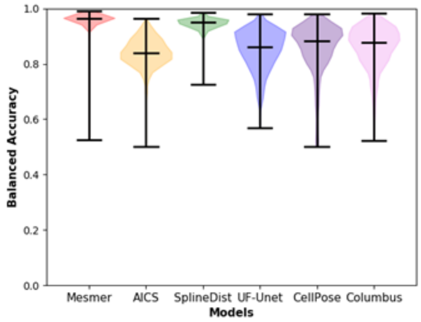

173

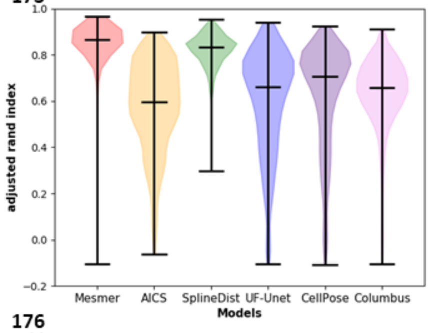

174

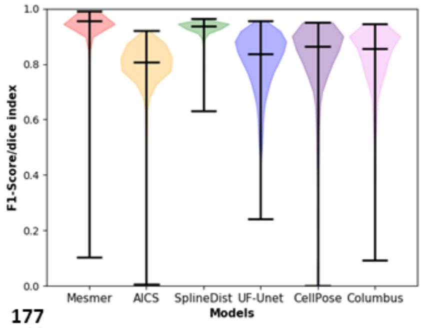

175

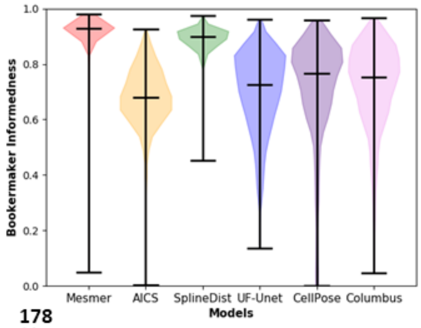

176

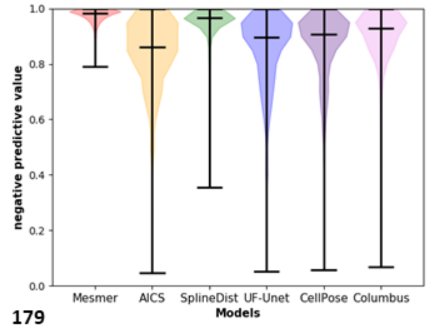

177

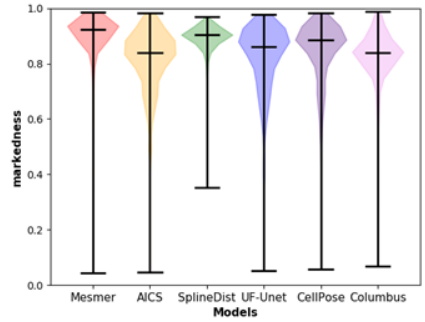

178

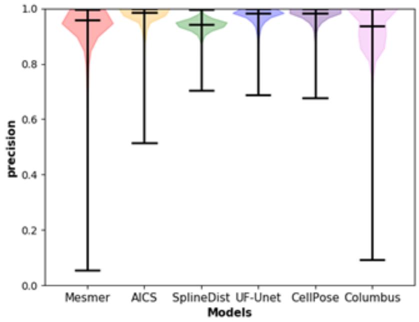

179

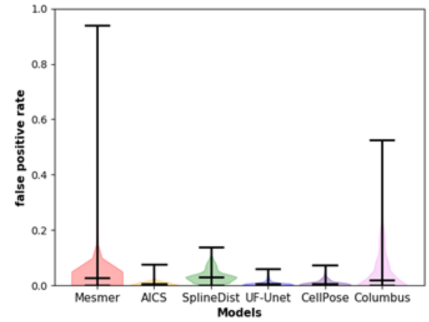

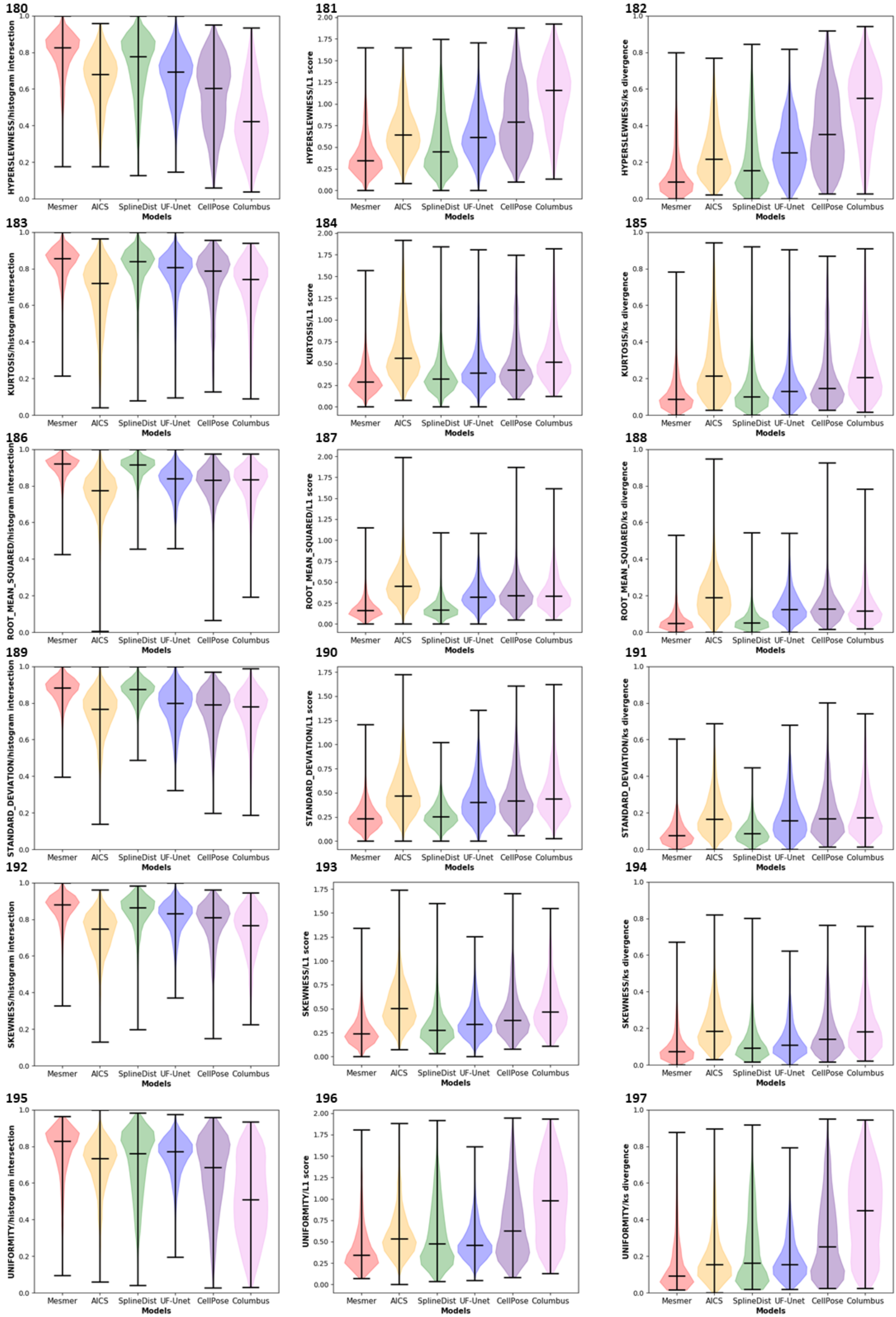

180
HYPERSLEWNESS/histogram intersection
181
HYPERSLEWNESS/L1 score
182
HYPERSLEWNESS/ks divergence
183
KURTOSIS/histogram intersection
184
KURTOSIS/L1 score
185
KURTOSIS/ks divergence
186
ROOT_MEAN_SQUARED/histogram intersection
187
ROOT_MEAN_SQUARED/L1 score
188
ROOT_MEAN_SQUARED/ks divergence
189
STANDARD_DEVIATION/histogram intersection
190
STANDARD_DEVIATION/L1 score
191
STANDARD_DEVIATION/ks divergence
192
SKEWNESS/histogram intersection
193
SKEWNESS/L1 score
194
SKEWNESS/ks divergence
195
UNIFORMITY/histogram intersection
196
UNIFORMITY/L1 score
197
UNIFORMITY/ks divergence
Mesmer
AICS
SplineDist
UF-Unet
CellPose
Columbus
Models

198

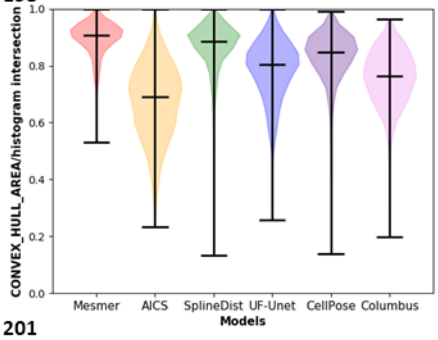

199

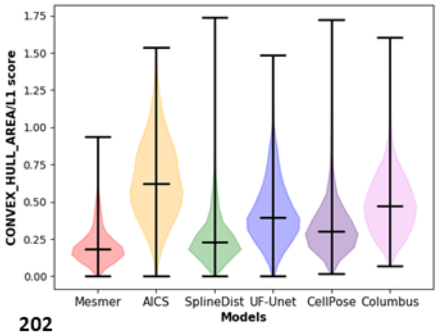

200

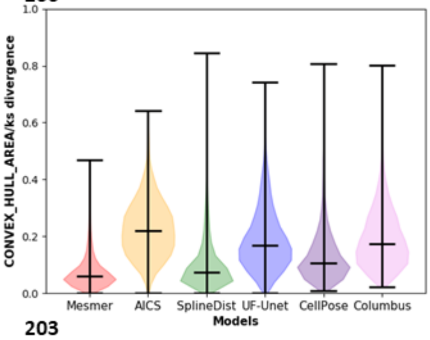

201

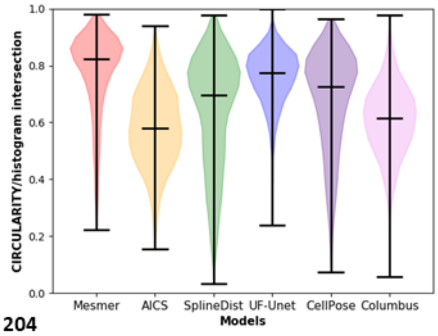

202

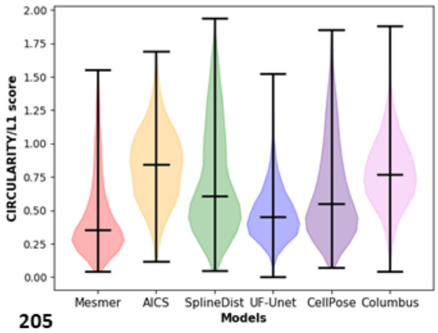

203

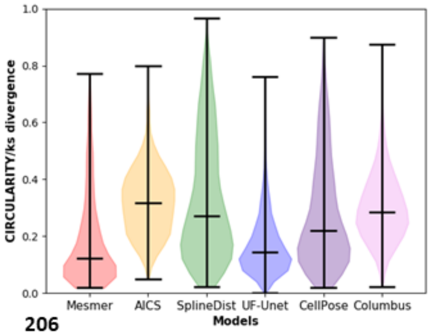

204

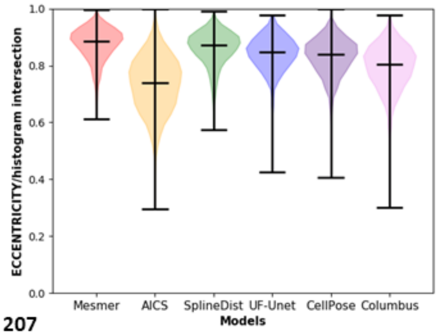

205

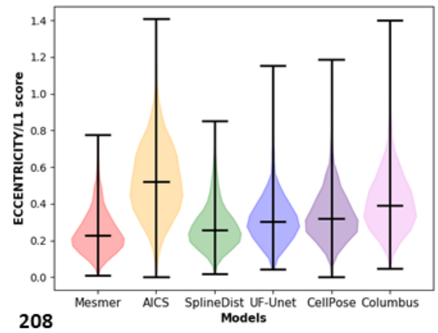

206

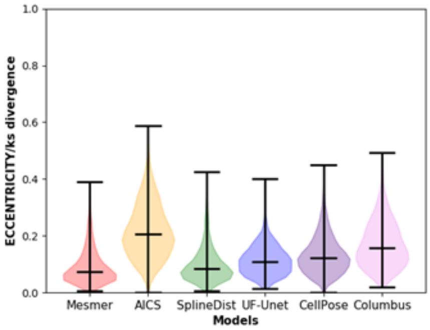

207

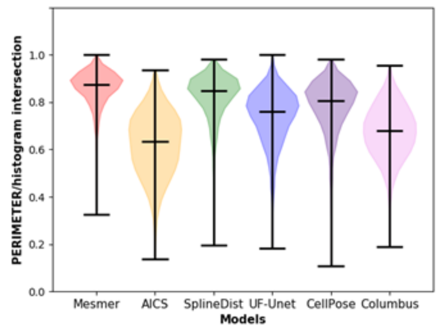

208

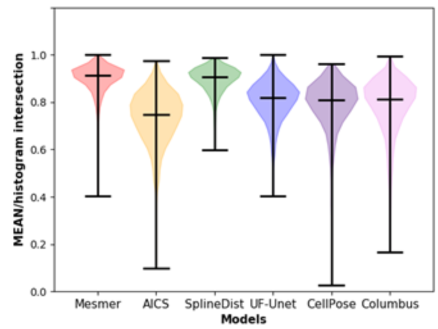

209. Eigenvectors (Absolute coefficient values from PCA) for ROI Level Metric Selection for Nuclear Segmentation using Pre-trained Models.

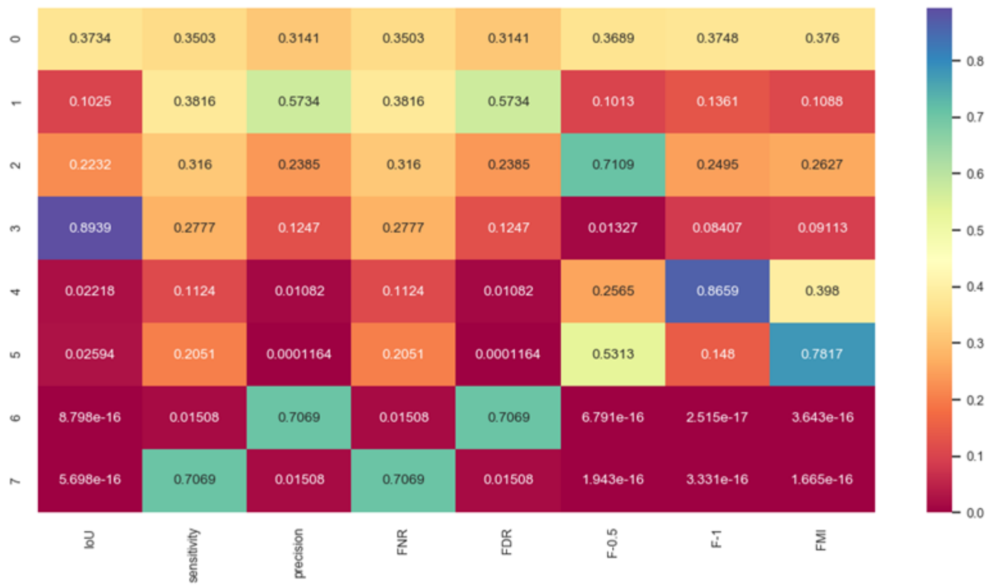

210. Pixel level Metric Selection for Nuclear Segmentation using Pre-trained Models **I.** Percentage Variance (Eigenvalue) **II.** Eigenvalue multiplied by Eigenvector. III. Absolute Coefficient Values from PCA (Eigenvector).

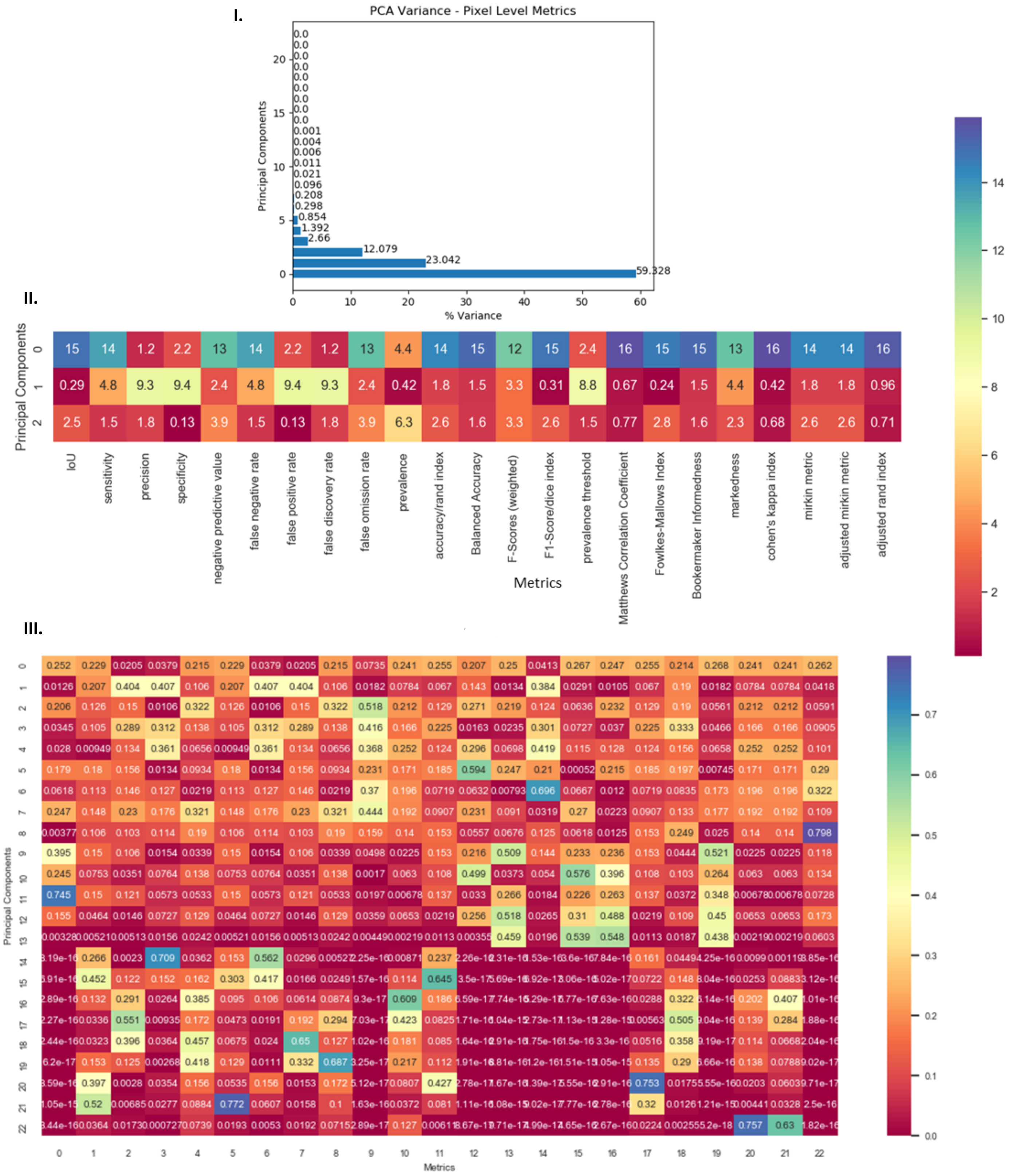

211. Feature level Metric Selection for Nuclear Segmentation using Pre-trained Models **I.** Percentage Variance (Eigenvalue) **II.** Eigenvalue multiplied by Eigenvector. III. Absolute Coefficient Values from PCA (Eigenvector).

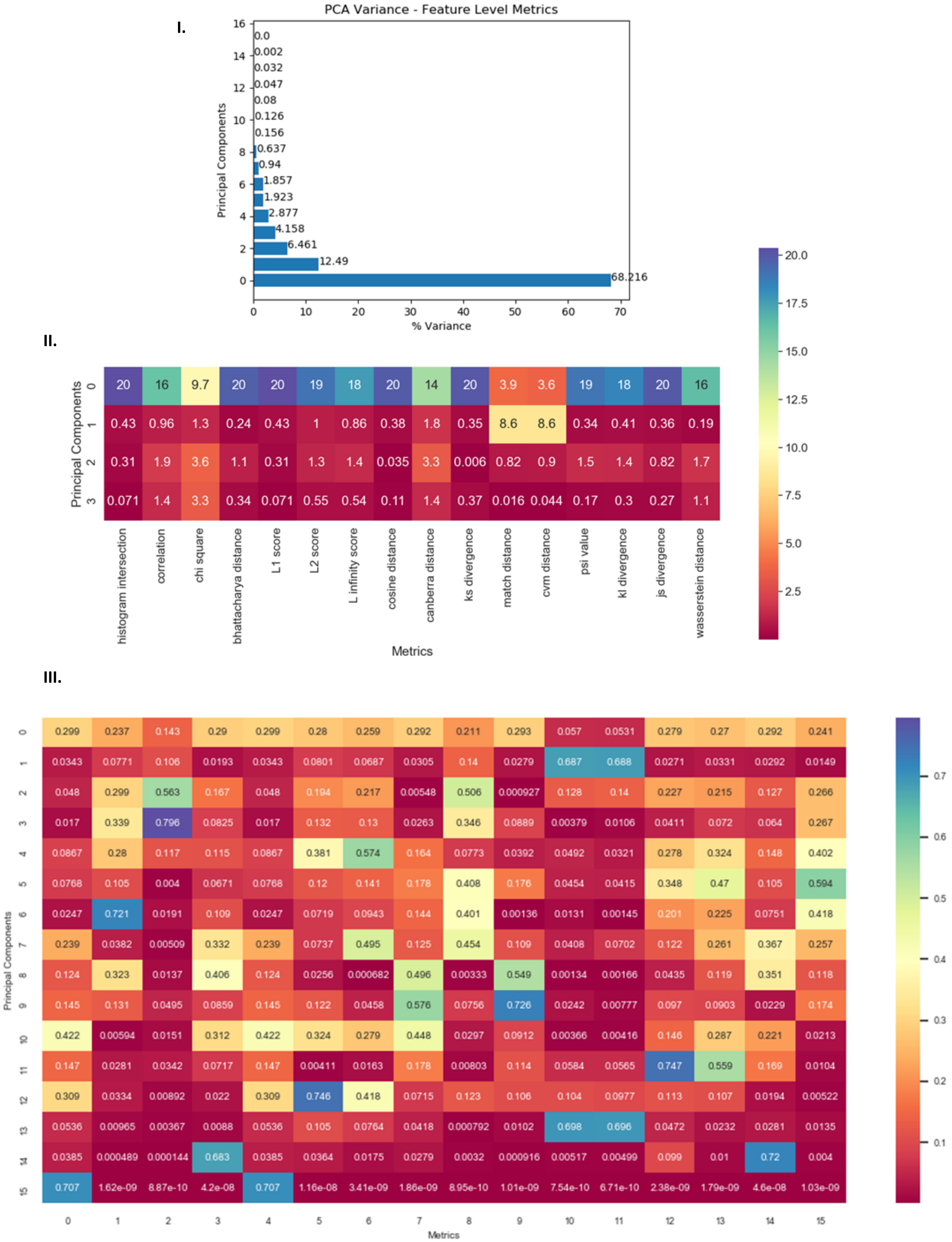

212. ROI level Metric Selection for Cytoplasm Segmentation using Pre-trained Models **I.** Percentage Variance (Eigenvalue) **II.** Eigenvalue multiplied by Eigenvector. III. Absolute Coefficient Values from PCA (Eigenvector).

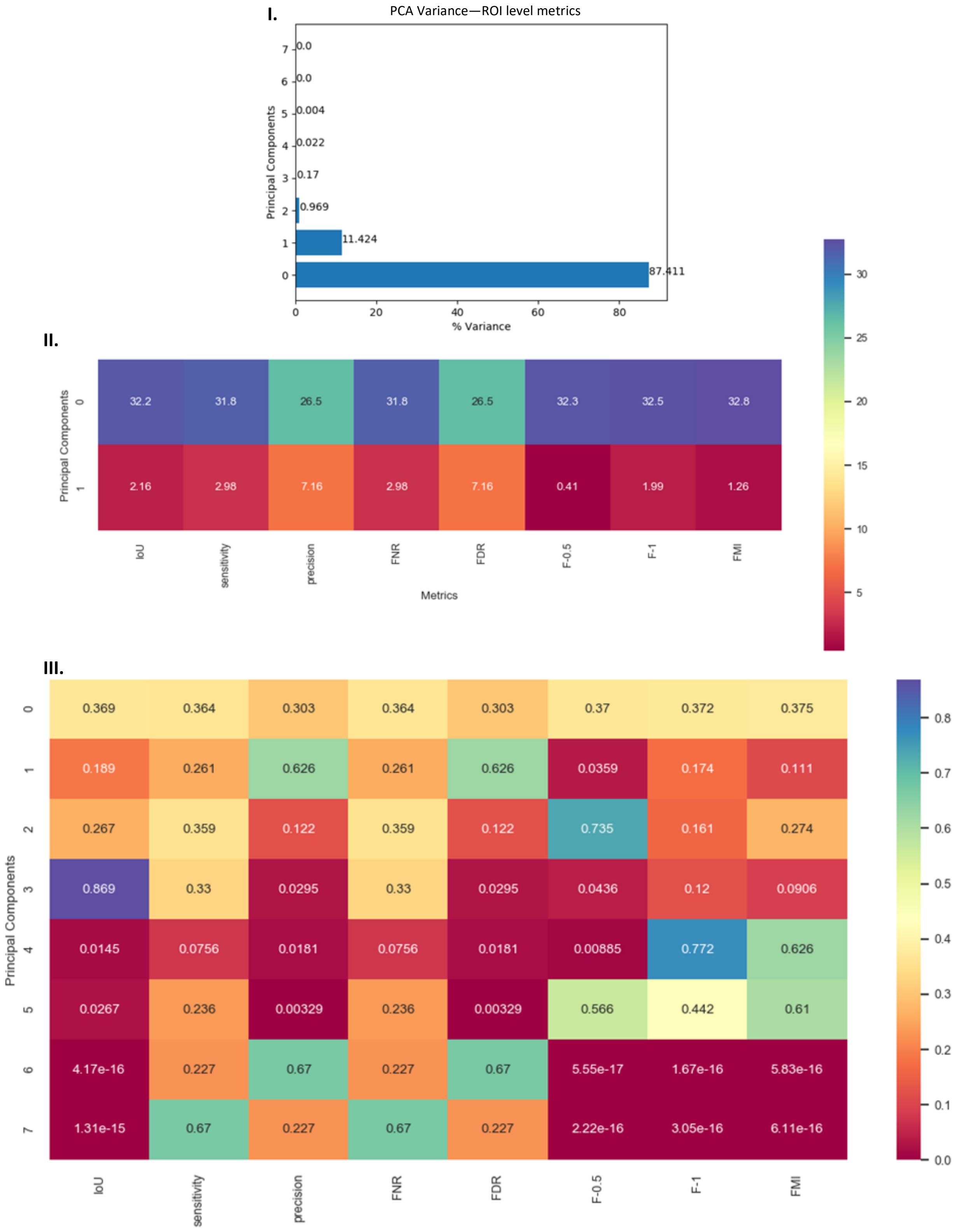

213. Pixel level Metric Selection for Cytoplasm Segmentation using Pre-trained Models **I.** Feature Importance from Catboost Classifier **II.** Percentage Variance (Eigenvalue) **III.** Eigenvalue multiplied by Eigenvector. **IV.** Absolute Coefficient Values from PCA (Eigenvector).

I.

Feature Id	Feature Importance
1	17.54072940744766
5	17.200146202015418
17	8.065305130913288
11	7.850149993542754
15	4.526859796625414
22	4.419694604414832
16	4.252057897489361
13	4.169450396936251
19	3.9410314275061773
0	3.555406335088331
2	2.8351663780422536
7	2.833880471386153
6	2.607928317523708
12	2.1707470075113626
3	1.8977039238167108
8	1.8412911792991449
18	1.7974374432295392
21	1.7798895395125636
20	1.5995092378019795
9	1.3672113240976154
10	1.2962264035322775
4	1.2473095172903115
14	1.2048680649769363

II.

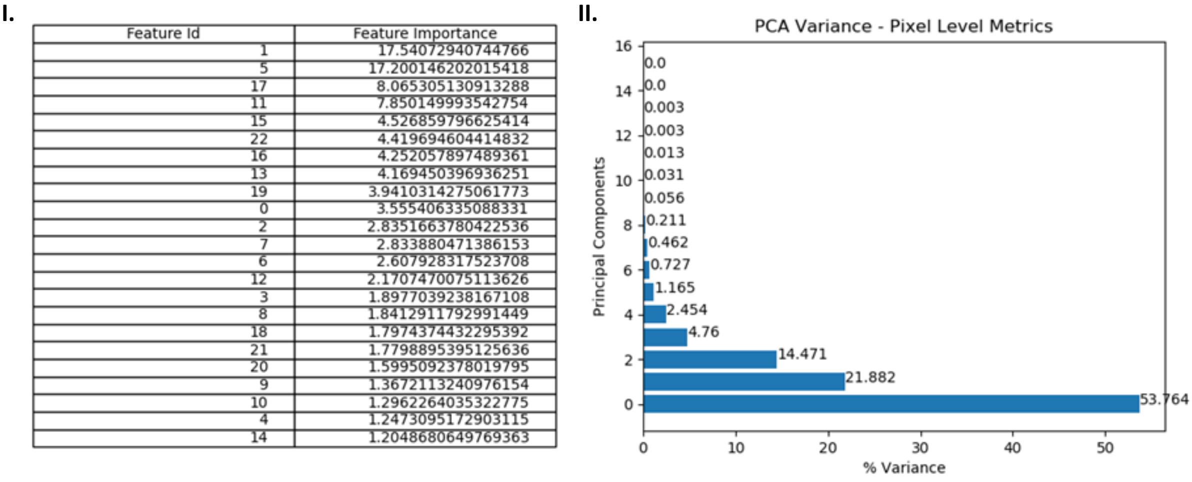

III.

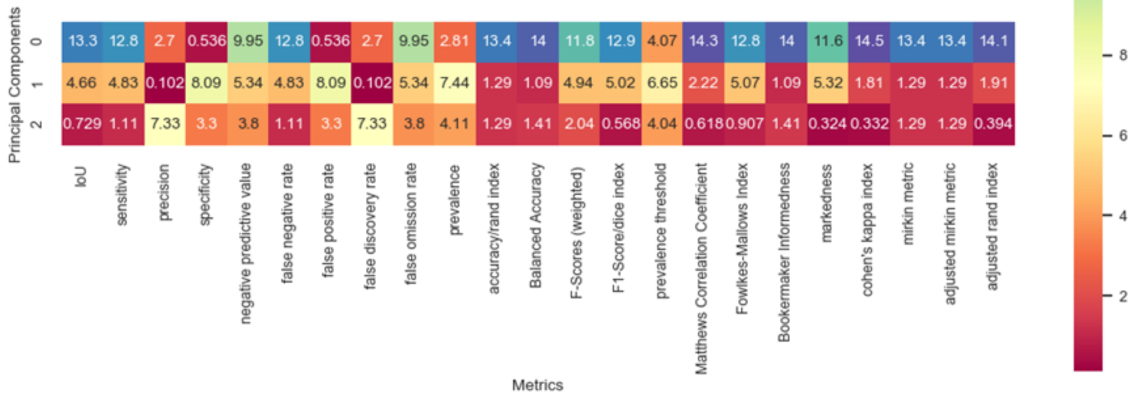

IV.

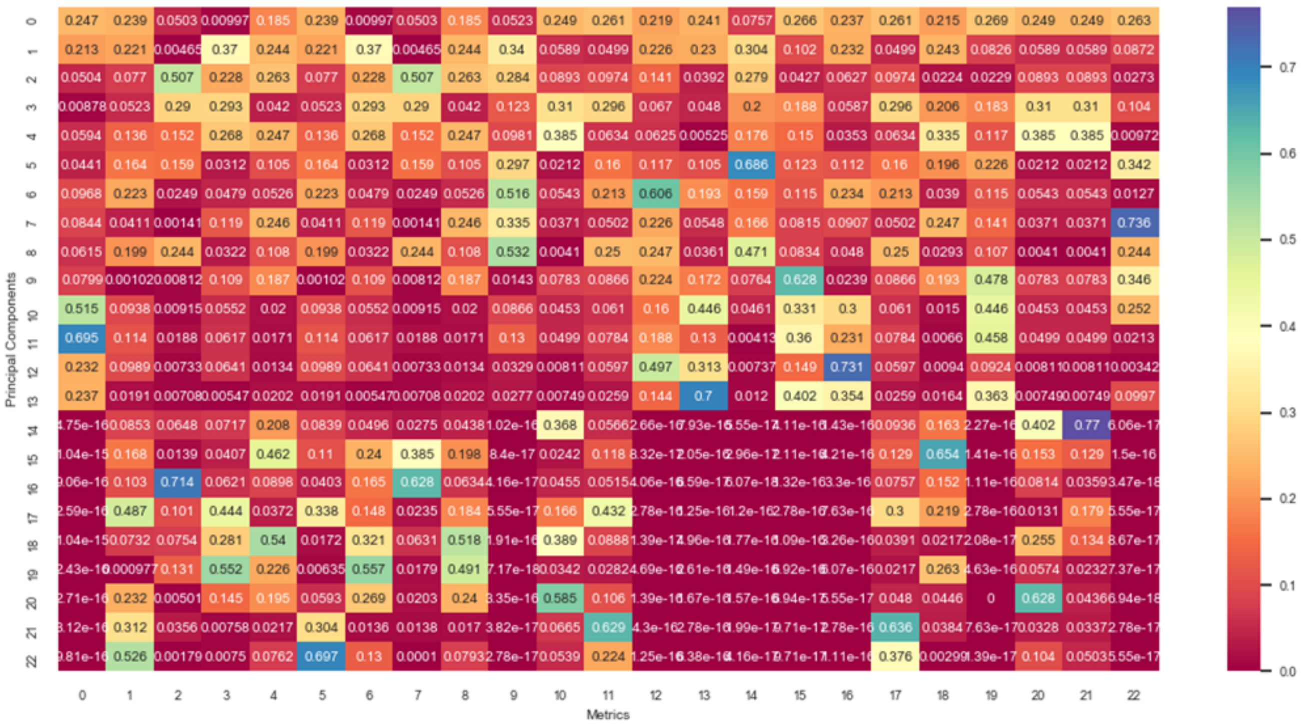

214. Feature level Metric Selection for Cytoplasm Segmentation using Pre-trained Models **I.** Percentage Variance (Eigenvalue) **II.** Eigenvalue multiplied by Eigenvector. III. Absolute Coefficient Values from PCA (Eigenvector).

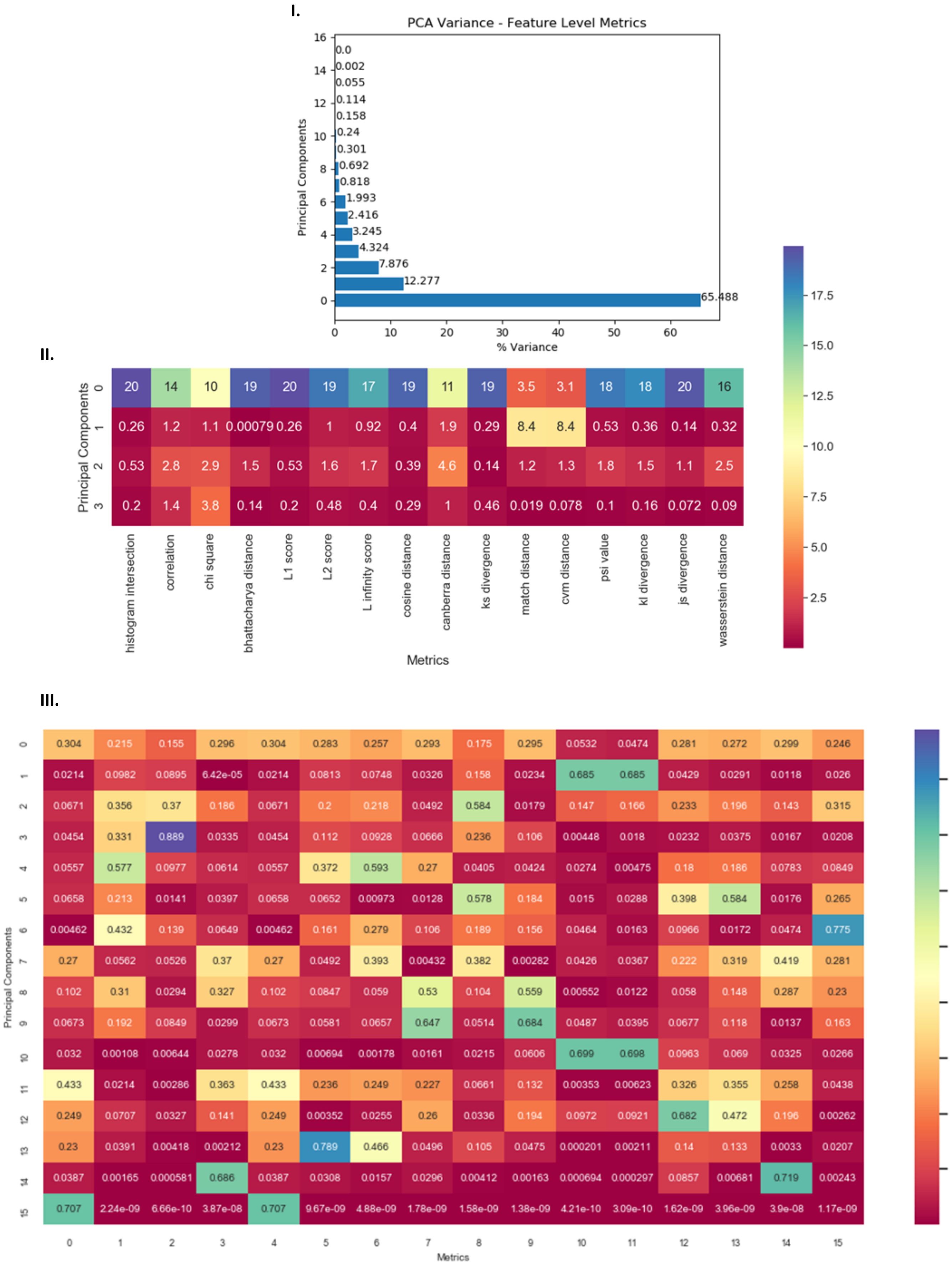

Supplement: Supplementary file 1 — Additional file 1: 207 region, pixel, and feature comparison graphs, PCA and CatBoost feature importance metrics, across both cell nuclei and cytoplasm. [file 12859_2023_5486_MOESM1_ESM.pdf]
