# Supplementary material for: Unbiased image segmentation assessment toolkit for quantitative differentiation of state-of-the-art algorithms and pipelines
Source: BMC Bioinformatics. 2023 Oct 12;24:388. doi: 10.1186/s12859-023-05486-8 (PMC10568754; doi:10.1186/s12859-023-05486-8)
Supplement: Supplementary file 2 — Additional file 2: Formula for all extracted metrics. [file 12859_2023_5486_MOESM2_ESM.pdf]

Metrics for Object and Pixel Level Comparison

$$IoU = \frac{TP}{TP + FP + FN}$$

$$Sensitivity\ or\ True\ Positive\ Rate\ (TPR) = \frac{TP}{TP + FN}$$

$$Precision = \frac{TP}{TP + FP}$$

$$Specificity\ or\ True\ Negative\ Rate\ (TNR) = \frac{TN}{TN + FP}$$

$$False\ Negative\ Rate = \frac{FN}{TP + FN}$$

$$False\ Postive\ Rate = \frac{FP}{TN + FP}$$

$$False\ Discovery\ Rate = \frac{FP}{TP + FP}$$

$$False\ Omission\ Rate = \frac{FN}{TN + FN}$$

$$F0.5\ Score = \frac{(1 + 0.5^2) * TP}{(1 + 0.5^2) * TP\ +\ (0.5^2) * FN\ +\ FP}$$

$$F1\ Score\ or\ Dice\ Index = \frac{(2 * TP)}{2 * TP\ +\ FN\ +\ FP}$$

$$Fowlkes - \ Mallows\ Index = \frac{TP}{\sqrt{(TP\ +\ FP) *\ (TP +\ FN)}}$$

$$Negative\ Predictive\ Value = \frac{TN}{TN + FN}$$

$$Prevalence = \frac{TP + FN}{TOTAL}$$

$$Accuracy\ or\ Rand\ Index = \frac{TP + TN}{TOTAL}$$

$$Balanced\ Accuracy = \frac{0.5 * TP}{TP + FN} + \frac{0.5 * TN}{TN + FP}$$

$$Prevalence\ Threshold = \frac{TNR - 1 + \sqrt{TPR * (1 - TNR)}}{TPR + TNR - 1}$$

$$Matthews\ Correlation\ Coefficient = \frac{(TP * TN) - (FP * FN)}{\sqrt{(TP + FP) * (TP + FN) * (TN + FN) * (TN + FP)}}$$

$$Bookermaker\ Informedness = \frac{TP}{TP + FN} + \frac{TN}{TN + FP} - 1$$

$$Markedness = \frac{TP}{TP + FP} + \frac{TN}{TN + FN} - 1$$

$$Expected\ Accuracy = \frac{(TP + FP) * (TP + FN) + (TN + FN) * (FP + TN)}{(TOTAL) * (TOTAL)}$$

$$Cohen's\ Kappa\ Index = \frac{Accuracy - Expected\ Accuracy}{1 - Expected\ Accuracy}$$

$$Mirkin\ Metric = (TOTAL)*(TOTAL) * (1 - Accuracy)$$

$$Adjusted\ Mirkin\ Metric = \frac{Mirkin\ Metric}{(TOTAL) * (TOTAL)}$$

$$Adjusted\ Rand\ Index = \frac{\left(\binom{TP}{2}+\binom{TN}{2}+\binom{FP}{2}+\binom{FN}{2}\right)-\frac{\left(\binom{TP+FP}{2}+\binom{FN+TN}{2}*\binom{TP+FN}{2}+\binom{FP+TN}{2}\right)}{\binom{TOTAL}{2}}}{0.5*\left(\binom{TP+FP}{2}+\binom{FN+TN}{2}+\binom{TP+FN}{2}+\binom{FP+TN}{2}\right)-\frac{\binom{TP+FP}{2}+\binom{FN+TN}{2}*\binom{TP+FN}{2}+\binom{FP+TN}{2}}{\binom{TOTAL}{2}}}$$

Where,

$TP\ =\ True\ Positive, TN\ =\ True\ Negative, FP\ =\ False\ Positive, FN\ =\ False\ Negative$ and $TOTAL\ =\ TP\ +\ TN\ +\ FP\ +\ FN$

Distribution Metrics for feature comparison

$$L1\ or\ Manhattan\ Distance = \sum_I |H_1(I) - H_2(I)|$$

$$L2\ or\ Euclidean\ Distance = \sum_I (H_1(I) - H_2(I))^2$$

$$L\infty\ or\ Chybyshev\ Distance = max_I(|H_1(I) - H_2(I)|)$$

$$Kolmogorov - Smirnov\ Divergence = max_I(|C_1(I) - C_2(I)|)$$

$$Match\ Distance = \sum_I |C_1(I) - C_2(I)|$$

$$Cramer - von\ Mises\ Distance = \sum_I \left(C_1(I) - C_2(I)\right)^2$$

$$PSI\ Value = \sum_I \left(\left(H_1(I) - H_2(I)\right) * \log\frac{H_1(I)}{H_2(I)}\right)$$

$$Kullback\text{–}Leibler\ Divergence\ (KLD) = \sum_I \left(\left(H_1(I)\right) * \log\frac{H_1(I)}{H_2(I)}\right)$$

$$Jensen\ Shannon\ Distance = 0.5 * KLD(H_1, (H_1 + H_2)/2) + 0.5 * KLD(H_2, (H_1 + H_2)/2))$$

Implemented using OpenCV functions

$$Histogram\ Intersection = \sum_I \min\left(H_1(I), H_2(I)\right)$$

$$Correlation = \frac{\sum_I (H_1(I) - \bar{H}_1)(H_2(I) - \bar{H}_2)}{\sqrt{\sum_I (H_1(I) - \bar{H}_1)^2 \sum_I (H_2(I) - \bar{H}_2)^2}}$$

$$Chi\ Square = \sum_I \frac{\left(H_1(I) - H_2(I)\right)^2}{H_1(I)}$$

$$Bhattacharya\ Distance = \sqrt{1 - \frac{\sum_I (H_1(I) . H_2(I))}{\sqrt{\bar{H}_1 . \bar{H}_2 . N^2}}}$$

Implemented using SciPy

$$Cosine\ Distance = 1 - \frac{H_1 . H_2}{||H_1||_2 ||H_2||_2}$$

$$Canberra\ Distance = \sum_I \frac{|H_1(I) - H_2(I)|}{|H_1(I)| + |H_2(I)|}$$

$$Wasserstein\ Distance = \int_{-\infty}^{\infty} |C_1 - C_2|$$

$Where,$

$H_1\ and\ H_2\ are\ probability\ distribution\ functions\ for\ ground\ truth\ and\ predicted\ features,$

$C_1\ and\ C_2\ are\ cumulative\ distribution\ functions\ for\ ground\ truth\ and\ predicted\ features,\ \bar{H}_k = \frac{1}{N}\sum_J H_k(J)$ and $N = Total\ Number\ of\ Histogram\ Bins$

Error Metrics

$$Mean\ Squared\ Error = \frac{\sum_I \left(H_1(I) - H_2(I)\right)^2}{TOTAL}$$

$$Root\ Mean\ Squared\ Error = \sqrt{\frac{\sum_I \left(H_1(I) - H_2(I)\right)^2}{TOTAL}}$$

$$Normalized\ Root\ Mean\ Squared\ Error = \frac{\sqrt{\frac{\sum_I \left(H_1(I) - H_2(I)\right)^2}{TOTAL}}}{\max(H_1(I)) - \min(H_1(I))}$$

$$Mean\ Error = \frac{\sum_I H_1(I) - H_2(I)}{TOTAL}$$

$$Mean\ Absolute\ Error\ (MAE)\ or\ Mean\ Absolute\ Deviation = \frac{\sum_I |H_1(I) - H_2(I)|}{TOTAL}$$

$$Geometric\ Mean\ Absolute\ Error = \mathrm{e}^{\frac{\sum_I \log(|H_1(I) - H_2(I)|}{TOTAL}}$$

$$Median\ Absolute\ Error = median(H_1(I) - H_2(I))$$

$$Percentage\ Error = \frac{H_1(I) - H_2(I)}{H_1(I)}$$

$$Mean\ Percentage\ Error = \frac{\sum_I \text{Percentage Error}}{TOTAL}$$

$$Mean\ Absolute\ Percentage\ Error = \frac{\sum_I |\text{Percentage Error}|}{TOTAL}$$

$$Median\ Absolute\ Percentage\ Error = median(|\text{Percentage Error}|)$$

$$Symmetric\ Mean\ Absolute\ Percentage\ Error = \frac{\sum_I 2.0 * \frac{|H_1(I) - H_2(I)|}{|H_1(I)| + |H_2(I)|}}{TOTAL}$$

$$Symmetric\ Median\ Absolute\ Percentage\ Error = median(2.0 * \frac{|H_1(I) - H_2(I)|}{|H_1(I)| + |H_2(I)|})$$

$$Mean\ Arctangent\ Absolute\ Percentage\ Error = \frac{\sum_I \arctan(|\text{Percentage Error}|)}{TOTAL}$$

$$Normalized\ Absolute\ Error = \sqrt{\frac{\sum_I (H_1(I) - H_2(I) - MAE)^2}{TOTAL_{H_1(I)} - 1}}$$

$$Normalized\ Absolute\ Percentage\ Error = \sqrt{\frac{\sum_I (Percentage\ Error - MAE)^2}{TOTAL_{H_1(I)} - 1}}$$

$$Root\ Mean\ Squared\ Percentage\ Error = \sqrt{\frac{\sum_I (Percentage\ Error)^2}{TOTAL}}$$

$$Root\ Median\ Squared\ Percentage\ Error = \sqrt{median((Percentage\ Error)^2)}$$

$$Integral\ Normalized\ Root\ Squared\ Error = \sqrt{\frac{\sum_I \left(H_1(I) - H_2(I)\right)^2}{\sum_I \left(H_1(I) - mean(H_1(I))\right)^2}}$$

$$Root\ Relative\ Squared\ Error = \sqrt{\frac{\sum_I \left(H_1(I) - H_2(I)\right)^2}{\sum_I \left(H_1(I) - mean(H_1(I))\right)^2}}$$

$$Relative\ Absolute\ Error = \frac{\sum_I |H_1(I) - H_2(I)|}{\sum_I |H_1(I) - mean(H_1(I)|}$$

$$Mean\ Directional\ Accuracy = \frac{\sum_I \sin(H_1(I)[1:] - H_1(I)[:-1]) == \sin(H_2(I)[1:] - H_2(I)[:-1])}{TOTAL}$$

Where,
H_1 and H_2 are probability distribution functions for ground truth and predicted feature, TOTAL is the number of samples and $TOTAL_{H_1(I)}$ *is the length of distribution* H_1.
