# Supplementary material for: Unbiased image segmentation assessment toolkit for quantitative differentiation of state-of-the-art algorithms and pipelines
Source: BMC Bioinformatics. 2023 Oct 12;24:388. doi: 10.1186/s12859-023-05486-8 (PMC10568754; doi:10.1186/s12859-023-05486-8)
Supplement: Supplementary file 3 — Additional file 3: Supplemenatary Tables 1–7. Tools and their github/dockerhub locations, and AI model performance as a function of sample size and training epochs. [file 12859_2023_5486_MOESM3_ESM.docx]

Supplementary Table 1: Plugins used in analysis pipelines as well as docker container location, and CWL tool (CLT) location.

| Version | Plugin | Github Repository | DockerHub Location |
| --- | --- | --- | --- |
| 0.1.7-GPU | UF – UNET Inference | https://github.com/PolusAI/polus-plugins-dl/tree/master/polus-unet-testing-plugin | labshare/polus-unet-testing-plugin |
| 0.2.4-GPU | UF – UNET Training | https://github.com/PolusAI/polus-plugins-dl/tree/master/polus-unet-training-plugin | labshare/polus-unet-training-plugin |
| 0.0.1 | SplineDist Inference | https://github.com/mmvih/polus-plugins/tree/splinedist-inference/polus-splinedist-inference-plugin | N/A |
| 0.0.1 | SplineDist Training | https://github.com/mmvih/splinedist/tree/training_big | N/A |
| 0.0.1 | CellPose | https://github.com/nishaq503/polus-plugins/tree/plugin/cellpose-inference/segmentation/polus-cellpose-inference-plugin | N/A |
| 0.0.6 | Mesmer Inference | https://github.com/Vishakha6/polus-plugins/tree/mesmerInference/segmentation/polus-mesmer-inference-plugin | polusai/mesmer-inference-plugin |
| 0.1.10 | AICS | https://github.com/PolusAI/polus-plugins/tree/master/segmentation/polus-aics-classic-seg-plugin | labshare/polus-aics-classic-seg-plugin |
| 0.2.1 | ROI Evaluation Pulgin | https://github.com/PolusAI/polus-plugins/tree/master/features/region-segmentation-eval | polusai/cellular-eval-plugin |
| 0.1.8 | Pixel Eval Plugin | https://github.com/PolusAI/polus-plugins/tree/master/features/pixel-segmentation-eval | polusai/pixelwise-eval-plugin |
| 0.1.9 | Feature Eval Plugin | https://github.com/PolusAI/polus-plugins/tree/master/features/feature-segmentation-eval | polusai/feature-eval-plugin |
| 0.2.4 | Nyxus | https://github.com/PolusAI/nyxus | polusai/nyxus |


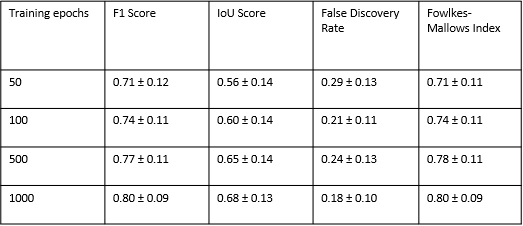

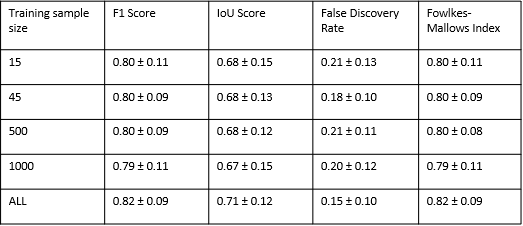
Supplementary Table 2 Supplementary Table 3


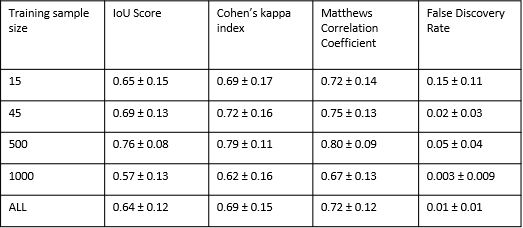

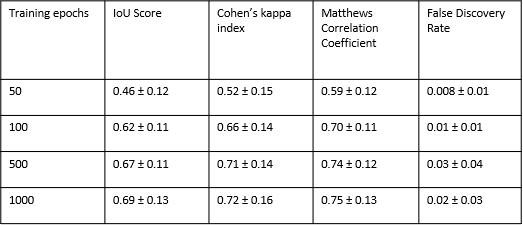
Supplementary Table 4 Supplementary Table 5


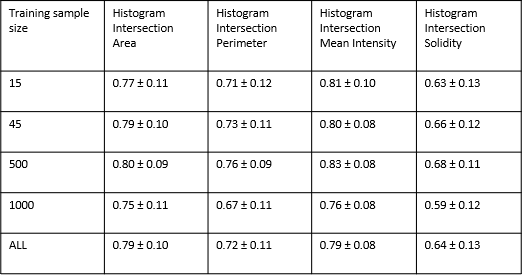

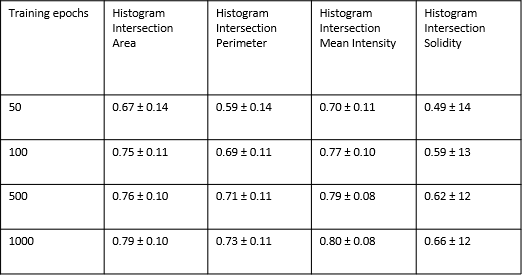
Supplementary Table 6 Supplementary Table 7
